# Arginase Structure and Inhibition: Catalytic Site Plasticity Reveals New Modulation Possibilities

**DOI:** 10.1038/s41598-017-13366-4

**Published:** 2017-10-19

**Authors:** Jérémie Mortier, Julien R. C. Prévost, Dominique Sydow, Sabine Teuchert, Christian Omieczynski, Marcel Bermudez, Raphaël Frédérick, Gerhard Wolber

**Affiliations:** 1 Drug Design Lab, Pharmaceutical & Medicinal Chemistry, Institute of Pharmacy, Freie Universität Berlin, Königin-Luise-Straße 2+4, Berlin, 14195 Germany; 2 Medicinal Chemistry Research Group, Louvain Drug Research Institute, Catholic University of Louvain, Avenue Mounier 73/B1.73.10, Woluwe-Saint-Lambert, 1200 Belgium

## Abstract

Metalloenzyme arginase is a therapeutically relevant target associated with tumor growth. To fight cancer immunosuppression, arginase activity can be modulated by small chemical inhibitors binding to its catalytic center. To better understand molecular mechanisms of arginase inhibition, a careful computer-aided mechanistic structural investigation of this enzyme was conducted. Using molecular dynamics (MD) simulations in the microsecond range, key regions of the protein active site were identified and their flexibility was evaluated and compared. A cavity opening phenomenon was observed, involving three loops directly interacting with all known ligands, while metal coordinating regions remained motionless. A novel dynamic 3D pharmacophore analysis method termed *dynophores* has been developed that allows for the construction of a single 3D-model comprising all ligand-enzyme interactions occurring throughout a complete MD trajectory. This new technique for the *in silico* study of intermolecular interactions allows for loop flexibility analysis coupled with movements and conformational changes of bound ligands. Presented MD studies highlight the plasticity of the size of the arginase active site, leading to the hypothesis that larger ligands can enter the cavity of arginase. Experimental testing of a targeted fragment library substituted by different aliphatic groups validates this hypothesis, paving the way for the design of arginase inhibitors with novel binding patterns.

## Introduction

Despite major advances in understanding the mechanisms leading to tumor immunity, a number of difficulties hinder the discovery of effective anti-tumor immunotherapies. Such obstacles include the ability of tumors to foster a tolerant microenvironment and the activation of a plethora of immunosuppressive mechanisms, which may synergistically act to prevent effective immune response^1^. Among others, one major mechanism is L-tryptophan catabolism by indoleamine 2,3-dioxygenase (IDO) or tryptophan 2,3-dioxygenase (TDO), frequently expressed in tumors. Recently published studies report that inhibiting these enzymes and thus rising L-tryptophan levels shows great potential for novel anti-tumor immunotherapy strategies^2–5^. Similar effects were observed for L-arginine levels, which are controlled by arginase in tumor cells^6^. However, in contrast to IDO and TDO, the only few known arginase inhibitors suffer from poor structural diversity. Therefore, the development of new arginase inhibitors has drawn considerable interest from medicinal chemists and pharmaceutical industry.

Arginase is a metalloenzyme occurring as two isoforms in human cells: Arginase1 (Arg1), in the cytosol, and Arginase2 (Arg2), in mitochondria^7^. Both catalyze the hydrolysis of L-arginine (L-Arg) to L-ornithine (L-Orn) and urea through a mechanism involving one hydroxide and two manganese ions^8^. Recent findings indicate that increased metabolism of L-Arg by myeloid derived suppressor cells producing Arg1 also inhibits T-lymphocyte response^6^. This enzyme is thus becoming a highly attractive target to fight cancer immunosuppression and cancer tumor growth^9–13^. Moreover, controlling intracellular arginase activity is highly interesting for the management of diseases associated with aberrant L-arginine homeostasis such as asthma^14–17^, erectile dysfunction^18–22^, cystic fibrosis^23^, and atherosclerosis^24–27^.

**Figure 1:**
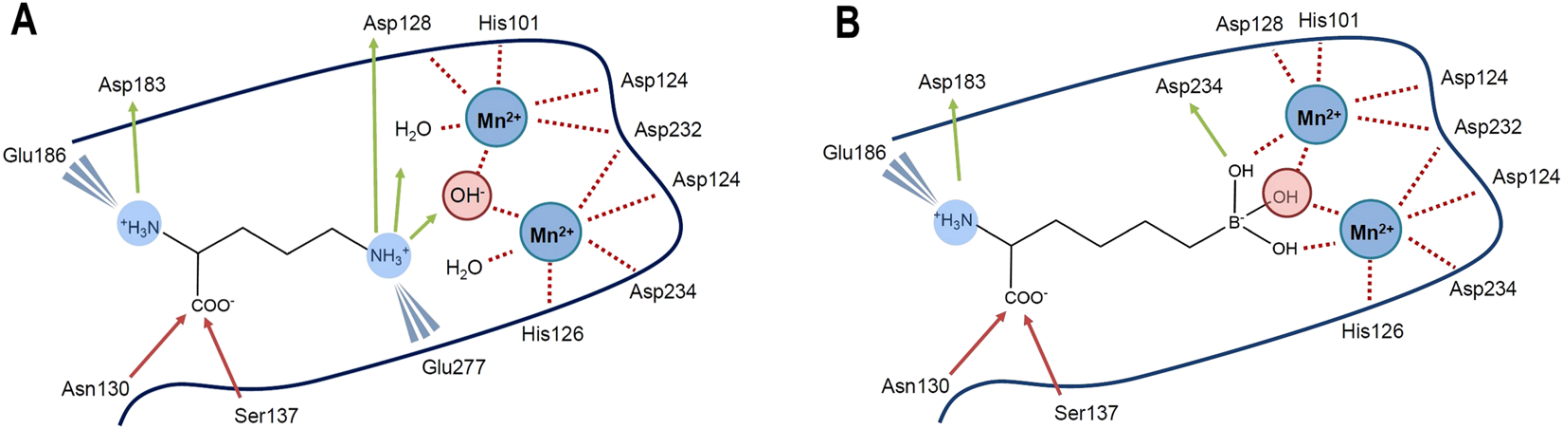
Comparison of the main ligand-arginase interactions for enzymatic product ornithine (left) and transition state analog ABH (right). Interactions color code: red/green arrows for H-bonds, and blue thorns for electrostatic interactions.

Two types of inhibitors are known to bind to arginase and they structurally only differ at the level of the group complexing the ion cluster. The first group comprises product analogues^28,29^, including compounds containing 2-aminoimidazole moieties^30^, and are stabilized by the hydroxide effectively bridging the ligand and the manganese ions (Fig. 1, left). In the case of the 2-aminoimidazole fragment, crystal structures indicate that this moiety does not displace the hydroxide but mimics its interaction with arginine, preventing the hydrolysis normally taking place with the natural substrate^30^. The second group comprises transition state analogues like hydroxyarginines^31–33^, boronic acids^20,29^, or sulfonamides^8^, which displace or react with the hydroxide group to directly coordinate the two manganese ions (Fig. 1, right). Boronic acids like 2-*S*-amino-6-boronohexanoic acid (ABH) and S-(2-boronoethyl)-l-cysteine (BEC) react with the hydroxide ion to form transition state analogs complexing the Mn^2+^ ions^34^. By displacing the metal-bridging hydroxide with a sulfonamide^8^, and the *N*-hydroxy^31^, respectively, L-amino acids S-(2-sulfonamidoethyl)-L-cysteine (SEC) and nor-N(ω)-hydroxy-l-arginine (nor-NOHA) also inhibit arginase by mimicking the transition state. Unfortunately, none of these inhibitors exhibit sufficient differences in affinity to provide isoform-selective inhibition in cultured cells or *in vivo* ^7,35^. Therefore, these known compounds represent preliminary lead structures, but the design of novel non-amino acid Arg1 inhibitors that selectively interact with Arg1 is a prerequisite for the development of novel Arginase1 modulators with therapeutic relevance^7^. The aim of this study is to combine computer-aided and experimental approaches to identify novel chemical structures for arginase inhibition. After an initial virtual screening campaign that did not yield new potent inhibitors, the arginase structure and its interaction with two representative ligands were carefully mechanistically studied from a conformationally dynamic perspective.

## Results and Discussion

### Virtual screening

A structural analysis of all ligand-enzyme interaction in the catalytic pocket of Arg1 was conducted using all structural data available in the Protein Data Bank (PDB). Two distinct domains of the catalytic cavity were distinguished: (a) the region binding to the L-amino acid moiety shared by the substrate, product and all known potent arginase inhibitors (interactions with Asp183 and Ser137) and, (b) the ion cluster stabilized by His101, Asp124, His126, Asp128, Asp232 and Asp234 (Fig. 1). A 3D-pharmacophore model describing key interactions identified for the binding of a transition state analogue was constructed (see supporting information for detailed methods). This model was used to screen databases of commercial compounds, leading to the selection of 19 compounds, purchased and tested experimentally in a radiometric assay^36^. At a concentration of 1 mM, no compound could inhibit more than 30% of arginase activity compared to full inhibition observed for BEC, used as reference inhibitor (Figure S1.2).

Our experience in virtual screening experiments is that negative results are observed only on rare occasions and that a carefully conducted pharmacophore-based VS campaign generally leads to the identification of inhibitors, even if sometimes weak in potency. Numerous examples have been published over the past 10 years by our group and others^37^. In this case, no significant activity was observed, although all selected compounds show excellent 3D-alignment with features extracted from validated inhibitors. Analyzing this result in detail is therefore important to better understand mechanisms of arginase inhibition.

The most trivial explanation for the lack of affinity of most molecules for Arg1 is that a strong binder is required to displace the hydroxide and replace it in the manganese coordination network. Nevertheless, the arginase pocket size is another critical point. Unlike proteins with large open binding clefts such as enzyme hydrolyzing peptides^38,39^, or carbohydrates^40,41^, or deep and complex active sites such as kinases^42,43^, or phosphatases^44^, crystal structures of arginase cavity indicate a small volume, allowing little structural diversity between the metal coordinating and the amino acid moiety of the ligand. In the design reported by Ilies *et al*.^30^, one fragment (2-aminoimidazole) binding to the ion cluster becomes 1000 times more potent when bound to a L-amino acid side chain (A1P), but completely loses this activity if one extra carbon is inserted between the two key interacting groups (A4P). These structural particularities confirm that designing potent arginase inhibitors requires high precision.

Nevertheless, it is interesting to notice that many of the molecules selected by the 3D pharmacophore model do not contain a three to four carbon aliphatic chain between the amino acid moiety and the metal complexing group (Figure S1.1), as observed for all known potent inhibitors. Even though the model failed to discover novel arginase inhibitors, the selection of several compounds with 5- and 6-membered rings as linker indicates that the geometry of the pocket could perhaps allow bulkier chemical groups to enter. Verifying this hypothesis would open new possibilities for the design of novel arginase inhibitors. However, since none of the tested molecules showed expected potency, a thorough investigation of the active site of Arg1 was first initiated using molecular dynamics simulations.

### Molecular dynamics

Molecular dynamics (MD) have become an essential technique used in contemporary molecular modeling to investigate macromolecular structures^45^, or their interactions with different binding partners^46,47^. Due to the major impact of protein flexibility on ligand binding, a study of the conformational variability of arginase was undertaken. With all-atoms systems solvated in explicit water (TIP3P)^48^, molecular simulations with three different systems were conducted: (i) the apo structure of Arg1, the ornithine-Arg1 complex and the ABH-Arg1 complex. Each system was simulated using Desmond^49^, with OPLS force field^50^, during 1 µs (5 × 200 ns) with a pressure of 1.01325 bars and a temperature of 300 K using a NPT ensemble (constant pressure, temperature and number of particles).

Analysis of the average deviation of Cα’s from the initial conformation (RMSD, Figure S2.1) indicates that the highest measured movements were observed for the apo enzyme with a value reaching a plateau around 3 Å for all simulations. Deviations observed with ligand-arginase complexes fluctuate between 1 Å and 3 Å. Interestingly, in case of the ABH-arginase complex, the observed movements of the loops in the area of the binding site indicate in some simulations a closure of the cavity around the inhibitor. Contrarily, trajectories of the ornithine-arginase complex show a relaxation of the active site within the first nanoseconds of simulation time. Analysis of the size of the pocket volume using Povme2.0^51^ illustrates the phenomenon of cavity opening observed with ornithine, while the same volume remains constant in the case of the apo enzyme (Figure S2.3). These results show that the presence of a ligand in the active site can induce a different effect on its surrounding protein structure.

**Figure 2:**
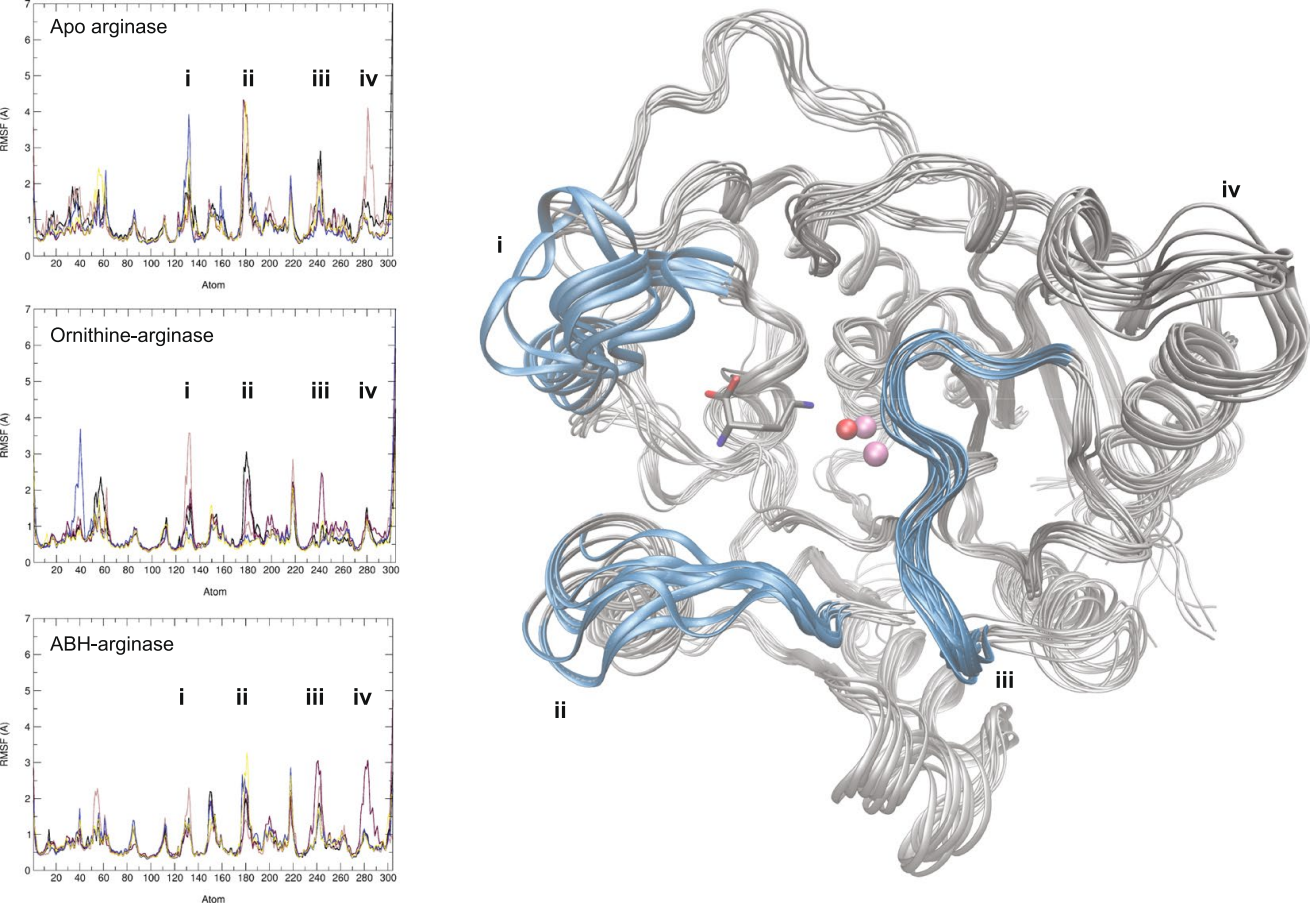
Root mean square fluctuations (RMSF) overlay for the five 200 ns repeats conducted with three different systems (left) and superposition of 10 protein conformations extracted from 200 ns simulation of the ornithine-Arginase 1 complex (right). The three flexible loops responsible for the opening of the binding pocket are highlighted in blue in the 3D view. Loops surrounding the active site cavity are labelled with roman numbers (**i**–**iv**). Mn atoms are represented in mauve and, the hydroxide group, in red. RMSF plots can be found in bigger format in supporting information.

After examination of the average fluctuations of all Cα (RMSF), the most dynamic regions of the protein structure were identified (Fig. 2). As expected, all systems displayed a high flexibility in regions at the surface of the protein and solvent exposed (loops including residues 212–225, 153–162 and 60–62), but also in the region of the binding site (loops 130–136, 175–183, and 240–246). The three main loops at the entrance of the cavity are (i) the region binding to the carboxylic acid of arginine (residues 130–136), (ii) the region binding to the neighboring primary amine (residues 175–183), and (iii) the loop forming the lid of the cavity above the ion cluster (residues 240–246). The orientation of these three key loops determinate whether the pocket entrance will be open or closed, as illustrated in Fig. 2 with the arginase-ornithine complex. Comparison of Cα fluctuations confirm larger movements with empty arginase as with arginase-ligand complexes. This observation is particularly true for loops directly interacting with the ligands, indicating a stabilization of the enzyme structure when bound to a small molecule. Contrarily, very little mobility is observed in the regions surrounding the ions cluster, *i.e*. residues 101, 124–128, and 232–234.

From the ligand perspective, we stressed earlier that substrate, product and potent inhibitors known to date show little to almost no chemical diversity. Yet, this study shows that loops delimitating the arginase binding site are highly flexible, suggesting high pocket volume plasticity. However, as all compounds previously identified by visual screening failed to efficiently bind to the enzyme, it appears essential to understand how known ligands behave in a dynamic cavity. To analyze and understand this, we studied ligand-enzyme interactions using 3D interaction patterns.

### Dynamic pharmacophores

To investigate and report in detail interactions involved in the stabilization of the studied ligands, a pharmacophore-based analysis method was developed. Since even local protein flexibility plays a major role in ligand recognition, we aimed at the development of an approach that can reflect conformational variability of the enzyme near the active site in a simple manner. Just like a fixed-in-time ligand-protein complex can be represented by a 3D-pharmacophore model^52^, identical recognition patterns in a dynamic environment can be collected as weighted interaction fields in a 3D-dynamic pharmacophore, or *dynophore*. The novelty of this approach is to integrate, in one single 3D-pharmacophore, all interactions detected throughout a complete MD simulation, including information on occurrence time and frequency. A previous version of this novel technique has been successfully applied to analyze subtle differences in ligand binding to muscarinic M2 receptors or HIV reverse transcriptase^53,54^.

**Figure 3:**
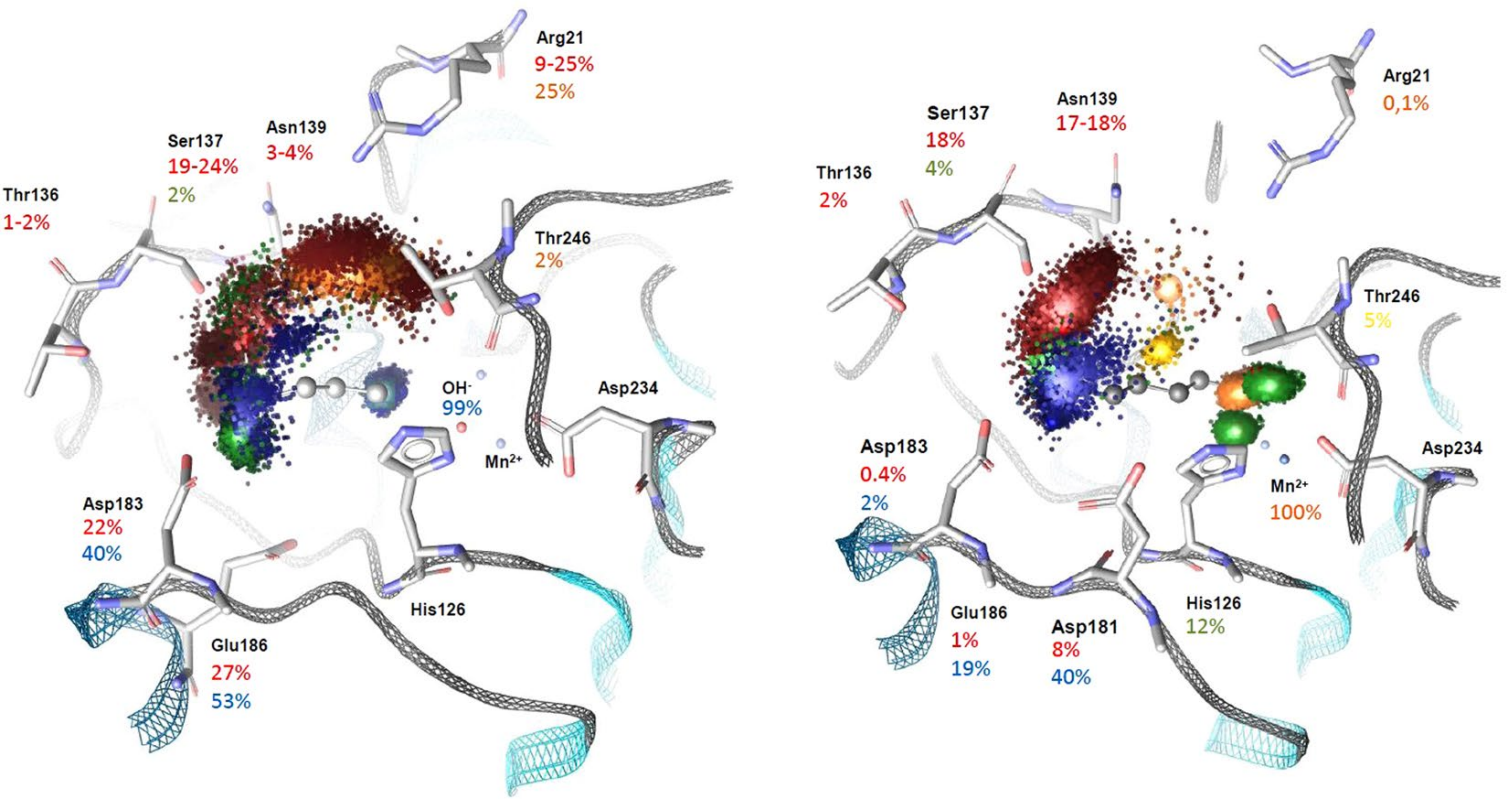
Dynophores constructed with frames from 1 µs MD simulation with enzymatic product ornithine (left) and inhibitor ABH (right). Color code: red for H-bond acceptor, orange for negative ionizable feature, green for H-bond donor, blue for positive ionizable moiety and yellow for hydrophobic contact. The manganese ions are represented by lavender spheres and the hydroxide group is in red.

For each frame of the resulting trajectories with the two arginase-ligand complexes, a point-based pharmacophore was generated and collected on a three-dimensional weighted interaction field. For each interaction, the frequency of its occurrence in the trajectory is illustrated in *bar codes*, while distance distributions are represented in histograms (Figure S3). A first dynophore field was built from all interactions detected in the MD simulation of Arg1 in complex with ornithine. This dynamic 3D-model clearly shows local flexibility in the arginase binding site and how the interaction pattern with a weak binder like ornithine fluctuates (Fig. 3). On one side of the cavity, H-bonds and coulomb interactions are detected during the entire simulation time between the hydroxide (with surrounding residues) and the primary amine on the side chain of L-ornithine. These interactions are represented by green (H-bond donor) and blue dots (positive charge) at the bottom of the cavity, in Fig. 3. On the other side, detected interactions involving the carbonate and amino groups of the amino acid moiety show a larger range of interaction partners, represented by a spread cloud of blue and red dots. Interactions involving the amino group are H-bonds (45.2% of simulation time) and electrostatic interactions (57.9% of simulation time). H-bond donor NH_3_^+^ interacts mostly with Asp183 and Glu186, when oriented as observed in its crystallized conformation^29^. The carboxylate group of ornithine interacts through electrostatic interactions (24.7%) as well as H-bonds (64.4% and 62.9% for each oxygen, respectively). Residues from loop 130–139 (mostly Ser137 and Asn130) are involved in the stabilization of the initial ligand conformation through H-bonds with the COO^−^ group. When the active site opens, residues further away like Thr246 and mostly Arg21 become the main interaction partners. To reach this region while maintaining an interaction with the ions cluster, the ligand has to operate a clockwise rotation around the hydroxide. We surmise that this movement, coupled with the opening of the cavity, is the initiating drive to expel the reaction product out of the arginase active site.

To confirm that the observed results are not biased by the computational method, the ornithine-arginase system was simulated using the Gromacs MD suite^51^ and Amber 99sb^55^ force field (method detailed in the experimental section). The same movement of the ligand was observed, *i.e*. rotation around the ion cluster and movement of the amino acid moiety towards the cavity exit stabilized through an interaction with Arg21. The generated dynophore and the detected interactions were very similar to those described above (data not shown for clarity).

In the dynophore resulting from 1 µs simulation of the ABH-arginase complex two main interacting groups are also clearly distinguished: The amino acid moiety and the boronate. Firstly, interactions involving the amino acid group are spread over a larger volume than with the boronate, confirming the flexibility of the enzyme in this region. Loops i (residues 130–139) and ii (residues 175–186) binding to the carboxylate and the amine, respectively, are able to operate a movement towards the solvent resulting in an opening of the cavity, as shown previously with ornithine (Fig. 2). The dynophore shows that the bound inhibitor ABH can follow this movement. The cloud of interactions detected in this region indicates the presence of H-bonds between the carboxylate of ABH and residues 130–139 during 36.8% to 36.9% of the simulation time (proportions measured separately for each oxygen atom). H-bonds were mostly involving Ser137, but also Asn130, Thr135, Thr136 and Asn139 (Figure S3.2). Interestingly, Thr246 and Arg21 are almost never reached by the COO^−^ group (<1%). Instead, the carboxylate of ABH is hold in the region of loop 130–139, allowing the ligand to conserve an ideal orientation for complexation of the ion cluster by the boronate. This major difference between the dynamic binding of ABH and ornithine is illustrated by an interaction cloud more concentrated around this particular loop for ABH (Fig. 3). Secondly, the amino moiety interacts as a positive ionizable feature. Charge interactions were detected for 26.5% of simulation time with residues Glu186, Asp183 and Asp181. H-bonds stabilizing the amino group were detected less frequently (7.6% of the analyzed frames, mostly with Ser137). Finally, the boronate is depicted in the dynophore as 3 H-bonds donors at the level of the 3 hydroxyl groups as well as one H-bond acceptor and one negative ionizable feature on the boron atom. These features illustrate the tight cloud of interactions detected between the boronate and the neighboring partners (arginase residues and manganese ions), indicating very little flexibility in this region, which is in agreement with a strong anchoring of the ligand at the bottom of the cavity.

Interestingly, hydrophobic contacts with the side chain of Thr246 were detected at the level of the aliphatic chain of ABH that links the amino acid to the boronic acid. Even if observed sporadically (5.2% of simulation time), the detection of this interaction indicates the possibility of creating hydrophobic contacts between the ligand and the enzyme in this particular region, despite a rather hydrophilic cavity.

### Fragment screening

This study of Arginase1 clearly gives a new insight on the arginase enzymatic cavity. The binding site shows a rigid architecture at the level of the ions cluster, and more flexibility in the region interacting with the amino acid moiety. Also, these results point out that the volume accommodating the chemical linker that connects the boronic acid of the inhibitors with its amino acid group is flexible. The consequence of this finding is that the enzymatic pocket can bear a chemical development of this specific part of the ligand. More than a simple chemical link, this fragment can become an actor of the ligand-enzyme binding, for example through hydrophobic contacts as observed in the region of Thr246 the with ABH. In order to consolidate our finding that larger molecules can enter the catalytic center of arginase and to identify chemical groups structurally different from trivial saturated carbon chains, a fragment-based approach was followed^52^.

**Figure 4:**
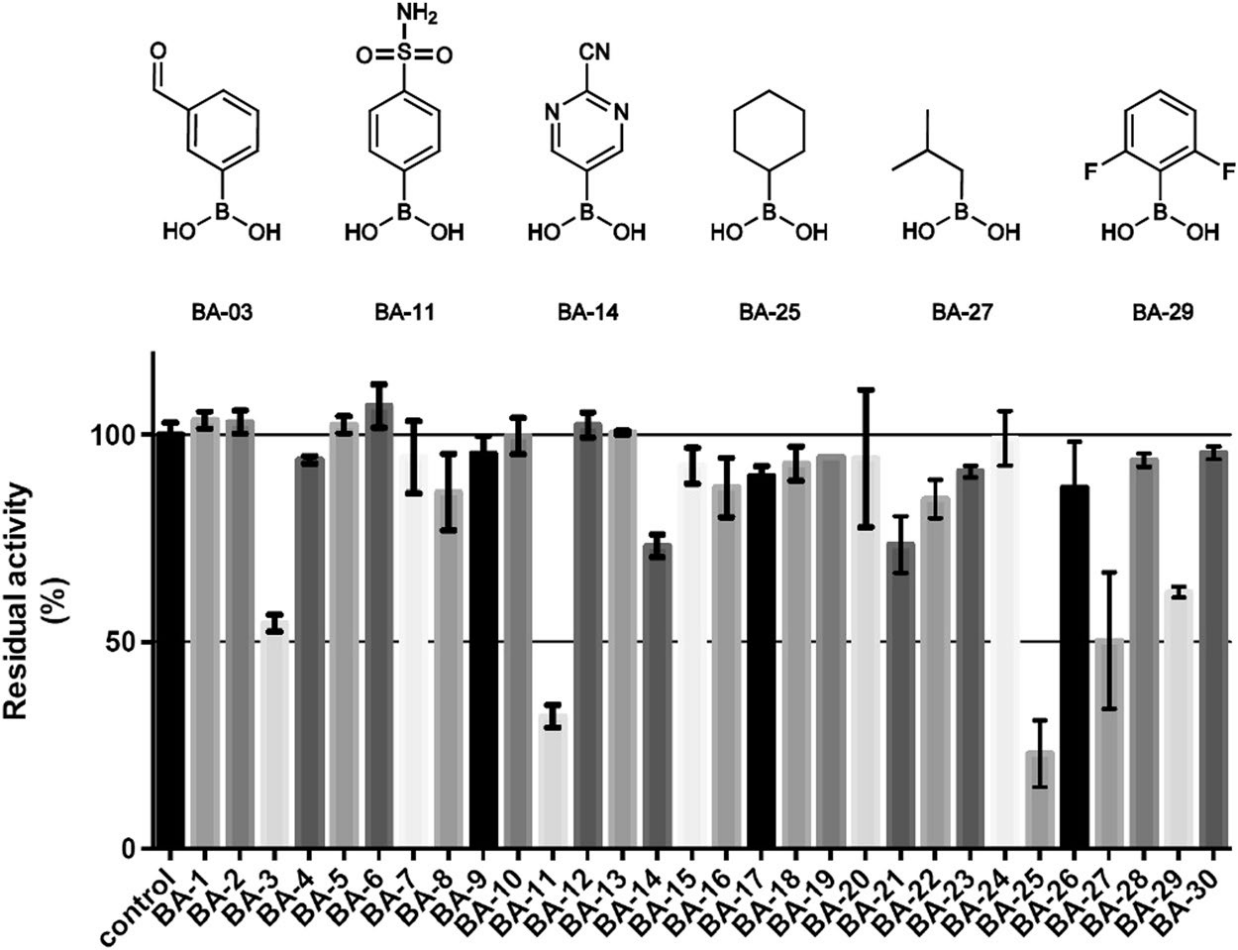
Residual arginase activity in presence of boronic acid fragments (concentration 1 mM) and chemical structure of the most potent compounds.

As the boronic acid group binds to the rigid region of the manganese ions, fragments functionalized by a boronic acid, but structurally diverse, were collected from vendors. Among all commercially available compounds identified, hydrophobic fragments with a larger volume than ABH were selected (e.g. 5- and 6-membered rings, see Figure S4.1). Then, to assess the ability of each fragments to be accommodated in this pocket, the inhibitory potency of all compounds was experimentally tested against Arginase 1. Out of 30 small fragments with a molecular mass ranging from 101 to 246 Daltons, two compounds inhibited more than 50% of arginase activity at a concentration of 1 mM (Fig. 4). Residual activity of arginase reached 35 ± 3% in presence of 4-sulfamoylbenzeneboronic acid (BA-11), while cyclohexylboronic acid (BA-25) showed the highest potency with 25 ± 4% residual activity.

This test reveals novel information about the active site of arginase: Firstly, the diminution of arginase activity in presence of a benzenoboronic compound clearly conveys new information on the flexibility of the cavity, even though the potency of this compound is low. This results indicates that benzenoboronic acid weakly binds to the enzyme and that a phenyl ring can enter the cavity. Despite being larger than a saturated carbon chain, cyclohexane displays a relatively good inhibitory potency considering the small size of this fragment (compound BA-25). Therefore, this result validates the hypothesis resulting from the MD study regarding the ability of this thin cavity to accommodate linkers larger than a saturated carbon chain.

To compare with results previously obtained with ornithine and ABH, MD simulations were carried out with cyclohexylboronic acid BA-25 for 1 µs and a dynophore model was generated (Figure S5.1). Predictably, stabilization of the boronate moiety operates following exactly the same pattern as observed with ABH. At the level of the cyclohexyl, hydrophobic interactions are detected with Thr246 during 31.7% of simulation time, which is six times more than in the ABH-arginase simulation. These results not only confirm the prediction that this hydrophobic fragment can enter the cavity of arginase, but also that stabilization of the ligand involves the same interaction partner as the one detected by MD with inhibitor ABH.

## Conclusion and Perspectives

This study is the first investigation of the arginase structure from a dynamic perspective. To rationalize a zero hit-rate virtual screening in a first round, MD simulations were carried out. This computationally driven investigation allowed comparing different regions of the protein active site with regard to their flexibility. Movement observed for three loops at the edge of the pocket were identified as a closing and opening phenomenon, while in comparison regions coordinating the ion cluster remained more static. A method based on dynamic pharmacophore (dynophore) was developed in order to analyze MD trajectories of ligand-enzyme complexes and to represent in a single 3D-model all interactions detected throughout a full simulation. This study highlights the flexibility of loops binding to the amino acid moiety of the ligand, directly affecting the size of the pocket where known inhibitors are binding. These observations could not have emerged from static arginase-ligand complexes. Ligand-enzyme interactions were described for the first time taking into account local conformational flexibility, and led to the hypothesis that ligands containing bulky moieties like phenyl or cyclohexane can enter the cavity of arginase. In order to experimentally confirm these hypotheses, 30 compounds were collected featuring a known metal complexing agent essential for binding arginase, and substituted by different aliphatic groups larger than the known saturated carbon chain. Measuring the inhibition potency of these compounds led to the identification of two novel fragments able to bind to arginase. These findings form the basis for the rational development of novel, non-amino acid arginase inhibitors.

The identified boronic acid fragments are not interesting as potential drugs to fight cancer, but are pharmacological tools that allowed us to validate the hypothesis generated from computational models. Working with a boronic acid moiety guaranteed a binding to the rigid part of the enzymatic pocket, while various fragments attached to the boronate allowed an exploration of the most flexible region identified by MD. We believe that the structural information reported in this study will inspire the design of the next arginase inhibitors and encourage medicinal chemists to consider protein flexibility in the frame of all structure-based drug design approach. Our work also clearly demonstrates that including protein binding site flexibility can unravel essential properties of an enzymatic pocket for a rational *dynamic* structure-based drug design. Therefore, we strongly believe that further application of the dynophore tool presented in this work can be extremely useful for structural biology, for in-depth intermolecular interaction analysis, and medicinal chemistry, for example to include dynamic information in pharmacophore-based virtual screening workflow.

## Methods

### Molecular dynamics

Crystal structures with the following code were retrieved from the Protein Data Bank (PDB): 3GMZ for the Arginase1-ornithine complex^29^, 2AEB for the arginase-ABH complex^34^, and 2PHA for the apo form of Arginase1^28^. The complex cyclohexyboronic acid-arginase was manually built from the 2AEB crystal structure, replacing the amino acid and aliphatic side chain of ABH by the cyclohexane of BA-25 using Molecular Operating Environment (MOE). All systems were prepared with the Maestro protein preparation tool to add hydrogens, remove the flexible and solvent exposed C-terminal loop 309-HKPIDYL-315, cap termini, and coordinate the ion cluster with zero bond order. Residues/atoms coordinating the first Mn^++^ are HIS126/ND1, ASP234/OD1 + 2, ASP124/OD1, ASP232/OD2 and the second Mn^++^ are ASP124/OD2, ASP128/OD2 HIS101/ND1 and also ASP232/OD2. Additionally, both manganese ions were coordinated by the charged oxygen of the hydroxide for the apo form and the complex with ornithine, or the complexing oxygen of the inhibitor, OH of boronate for ABH. Using Desmond (version 11.0.014) with OPLS2005 force field, the three selected enzyme-ligand complexes were placed in an orthorhombic box filled with about explicit TIP3P water molecules and neutralizing counter ions (Na+ or Cl−). The total amount of atoms reached between 31 K and 33 K for all systems. Minimization and equilibration was performed for each system using default parameters. Then, ligand-enzyme complexes were simulated for 200 ns with periodic boundary conditions. A pressure of 1,01325 bars was kept constant using the Martyna-Tobias-Klein barostat method^55^, and a temperature of 300 K was kept constant with a Nose-Hoover thermostat^56,57^. Short-range electrostatic interactions were calculated up to a distance of 9 Å cut-off. With RESPA integrator, constraints were used on all bonds with 2 fs time step length for bonded and near atoms, and 6 fs for far atoms. Each simulation was repeated five times and trajectories were concatenated before the dynophore analysis.

Using Gromacs (version 5.0.4-mpi,)^58^ with Amber 99 sb force field^59^, the ornithine-Arginase1 complex was solvated with explicit water molecules (TIP3P) in a 168 nm^3^ dodecahedron neutralized with Na^+^ and Cl^−^ counterions. Topology for ornithine was generated using Antechamber^60^ through the ACPYPE online platform^61^. Following steepest descent minimization, each complex was equilibrated for 0.1 ns using position restraints applied to all protein heavy atoms. Then each system was simulated for 200 ns (time step length = 2 fs), with periodic boundary conditions, using PME electrostatics (rcoulomb = 12 Å)^19^ and Van der Waals interactions cut-off (rvdw = 12 Å). Constraints were used on all bonds in the peptides (LINCS algorithm)^62^. The Nosé-Hoover coupling method was used (temperature of 300 K)^56,57^ together with the Parrinello-Rahman coupling method (pressure of 1 bar)^63^. The ion cluster coordination was controlled using distance restrains and the charges of Mn^+2^ and OH^−^ were set manually to + 2 and −1, respectively.

Gromacs was used for all RMSD, RMSF and cluster analysis using the g_rms, g_rmsf and g_cluster tools, respectively (alignments always performed using Cα for least squares fit and RMSD calculations)^58^. Pocket volume calculation was performed with Povme2.0^51^ using default parameters. The coordinates of the boron atom as center of a 10 Å-radius sphere defined the pocket cavity. All simulations were conducted on a GPU, locally and on the high-performance computing system SOROBAN of the Freie Universität Berlin (http://www.zedat.fu-berlin.de/Compute).

### Dynophores

The developed dynophore implementation automatically generates a pharmacophore for each frame of a given MD simulation and processes its pharmacophore features pursuing the following principle: Pharmacophore features detected during the simulation that are sharing the same interaction type (*e.g*. H-bond donor or hydrophobic contact) and the same ligand atoms are grouped into a so-called *superfeature*. The occurrence of each superfeature is monitored in terms of spatial, statistical and sequential behavior throughout the whole analyzed trajectory. (a) Spatial information: Dynophores are built by superimposing the 3D-pharmacophores of all trajectory frames aligned on the target Cα atoms. Subsequently, each feature is reduced to the coordinates at the centroid of the detected feature sphere and the resulting dynophore is represented by clouds of points in three dimensions. (b) Statistical information: The occurrence of each superfeature is monitored (*superfeature occurrence frequency*), whereby frequency is simply calculated relatively to the sum of all considered frames. Additionally, for each superfeature, all interaction with a partner on the target-side are also monitored (*interaction occurrence frequency*), whereby frequency is calculated relatively to the sum of the corresponding superfeature occurrences. Distance distributions are reported in histograms (*interaction distance histogram*) for each reported interaction. (c) Sequential information: Occurrence of superfeatures and their interactions are described sequentially in form of bar code series (*superfeature occurrence sequence* and *interaction occurrence sequence*) as well as interaction distances in form of distances series (*interaction distance sequence*). The dynophore program has been implemented within the ilib/LigandScout framework^64^.

### Enzymatic assay

Arginase inhibition was measured using a modified version of the fixed-point radioactive assay developed by Ruegg and Russell^36^. Assay mixtures contained 50 µL of 100 mM 2-(cyclohexylamino)ethanesulfonic acid (CHES)-NaOH (pH 9.0), 100 µM MnCl2, 0.05 µCi of [guanidino-14C]-L-arginine, 3 mM unlabeled L-arginine, and 5 µL of inhibitors in a 50 µL volume per centrifuge tube. Reactions were started by the addition of 5 µL of a10 µg/mL recombinant human Arginase1 solution. After 5 min, reactions were quenched by the addition of 400 µL of “stop” solution (0.25 mM acetic acid (pH 4.5), 7 M urea, 10 mM L-arginine, and a 1:1 (v/v) slurry of Dowex W-X8 in water). Each reaction mixture was immediately vortexed for 30 s after the addition of “stop” solution, and then centrifuged at 6000 rpm for 10 min. 200 µL of the supernatant was taken and 3 mL of scintillation liquid (Ultima Gold^tm^, PerkinElmer^®^) was added in preparation for liquid scintillation counting using a PerkinElmer^®^ counter (model: Tri-Carb 2900TR). The DPM value obtained for the negative control (containing no enzyme) was systematically subtracted. Results were expressed as percent of activity compared to the positive control (without inhibitor), after GraphPad Prism software was used to treat the data and to analyze the dose-response curves. Inhibitors potency was expressed as IC_50_ values. Reference inhibitor BEC was purchased from EnzoLifeSciences, Inc.

## Electronic supplementary material

Supplementary information
