# Supplementary material for: Arginase Structure and Inhibition: Catalytic Site Plasticity Reveals New Modulation Possibilities
Source: Sci Rep. 2017 Oct 19;7:13616. doi: 10.1038/s41598-017-13366-4 (PMC5648838; doi:10.1038/s41598-017-13366-4)
Supplement: Supplementary file 1 — Supplementary information [file 41598_2017_13366_MOESM1_ESM.pdf]

Arginase Structure and Inhibition: Catalytic Site Plasticity Reveals New Modulation Possibilities

Jérémie Mortier,[§] Julien Prévost,[#] Dominique Sydow,[§] Sabine Teuchert,[§] Christian Omieczynski,[§] Marcel Bermudez,[§] Raphaël Frédérick,[#]* Gerhard Wolber[§]*

[§] Group of Computer-aided Drug Design, Institute of Pharmacy, Free University of Berlin, Germany

[#] Medicinal Chemistry Research Group, Louvain Drug Research Institute, Catholic University of Louvain, Belgium

Corresponding authors: raphael.frederick@uclouvain.be, gerhard.wolber@fu-berlin.de

Supporting information

Abbreviations:

HBA = hydrogen bond acceptor

HBD = hydrogen bond donor

PI = positive ionizable

NI = negative ionizable

H = hydrophobic contact

1. Virtual screening

With a view to identify essential features required for an optimal ligand-enzyme interaction in the catalytic pocket of ARG1, all co-crystallized inhibitors available from the Protein Data Bank (PDB) were collected and 3D-pharmacophore models were created for each of them. Shared features from all models describing ligand-enzyme interactions where the hydroxide ion is displaced were selected and compiled in one pharmacophore model. The ability to discriminate between inhibitors and non-inhibitors) was assessed and improved using a dataset of 27 active compounds and 24 inactive compounds assembled from the literature, the Chembl database and in-house activity data. Multi-conformational screening of these compounds was carried out and the signal-noise ratio was controlled using a Receiver Operating Characteristics curve. After a stepwise iterative development, the final 3D-pharmacophore model was able to discriminate all inactive compounds and retain 23 of the active ones. A virtual screening (VS) of commercial libraries comprising over 106 drug-like compounds (from providers Asinex, Vitas M, Life Chemicals, Chembridge, Maybridge, Specs, Enamine and Prestwick) was conducted and the model picked 63 hits, among which the amino acids glutamate and asparagine. In the Prestwick database, levodopa and methyldopa were identified. Levodopa is dopamine receptors agonist used in the clinical treatment of Parkinson’s disease. Glutamic acid, asparagine, levodopa and methyldopa were not included in the final selection because of their expected low specificity. All retrieved hits were re-docked using GOLD and ranked based on the quality of their 3D-superposition with the pharmacophore model, using LigandScout. The best 19 compounds (Figure S1.1) were purchased and arginase residual activity was tested experimentally in presence of inhibitor using a radiometric assay measuring arginine consumption. At a concentration of 1mM, no compound could inhibit more than 30% of arginase activity compared to full inhibition observed for BEC, used as reference inhibitor (Figure S1.2).

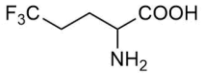

VS-01 // Maybridge BTB12855SC

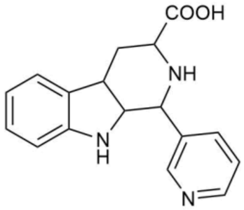

VS-02 // Asinex BAS00012703

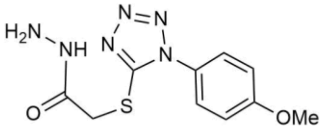

VS-03 // Asinex BAS02549930

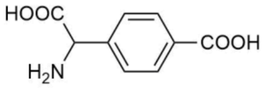

VS-04 // Life Chem F2167-0082

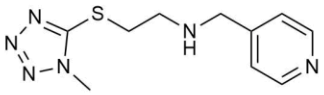

VS-05 // SPECS AN-465/42767370

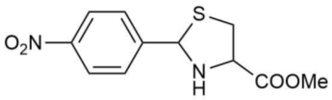

VS-06 // SPECS AJ-264/34031001

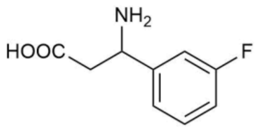

VS-07 // Enamine T0507-5484

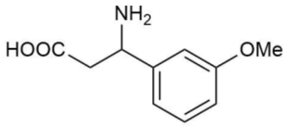

VS-08 // Enamine T6097003

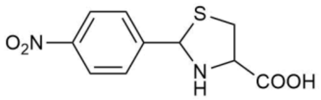

VS-09 // SPECS AF-399/33191035

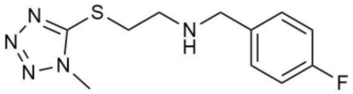

VS-10 // SPECS AN-465/42767543

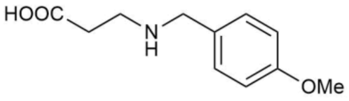

VS-11 // SPECS AN-465/43460962

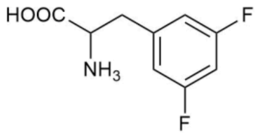

VS-12 // Maybridge SPB08033SC

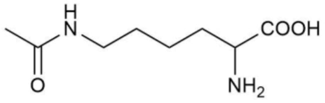

VS-13 // Maybridge BTB13551SC

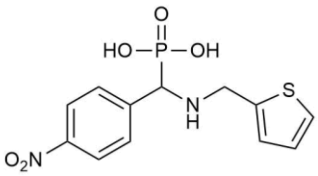

VS-14 // Enamine Z324265724

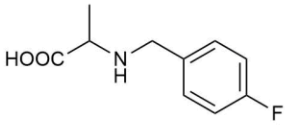

VS-15 // Enamine T6397618

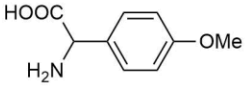

VS-16 // Enamine T5883816

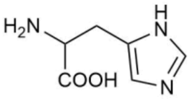

VS-17 // Enamine T5889146

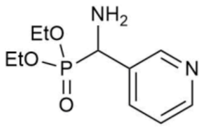

VS-18 // Enamine T0500-9868

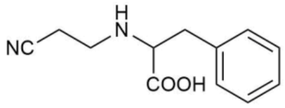

VS-19 // Enamine T5377051

Figure S 1.1: Molecules selected and tested after virtual screening on arginase 1

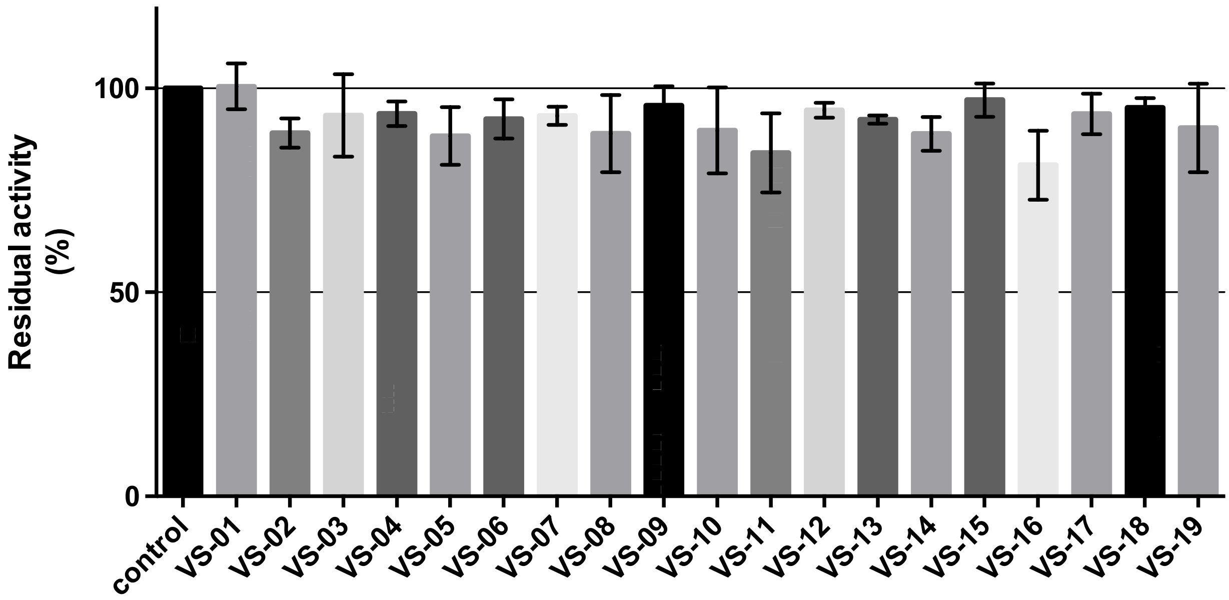

Figure S 1.2: Residual activity of arginase in presence of compounds selected by virtual screening at a concentration of 1 mM

2. MD simulations

The root mean square fluctuation to the starting conformation was monitored for all alpha carbons of the three simulated systems (Figure S 2.1). Using the same colour code, the root mean square fluctuations were also calculated for all alpha carbons (Figure S 2.2).

Apo enzyme

Ornithine-Arginase complex

ABH-Arginase complex

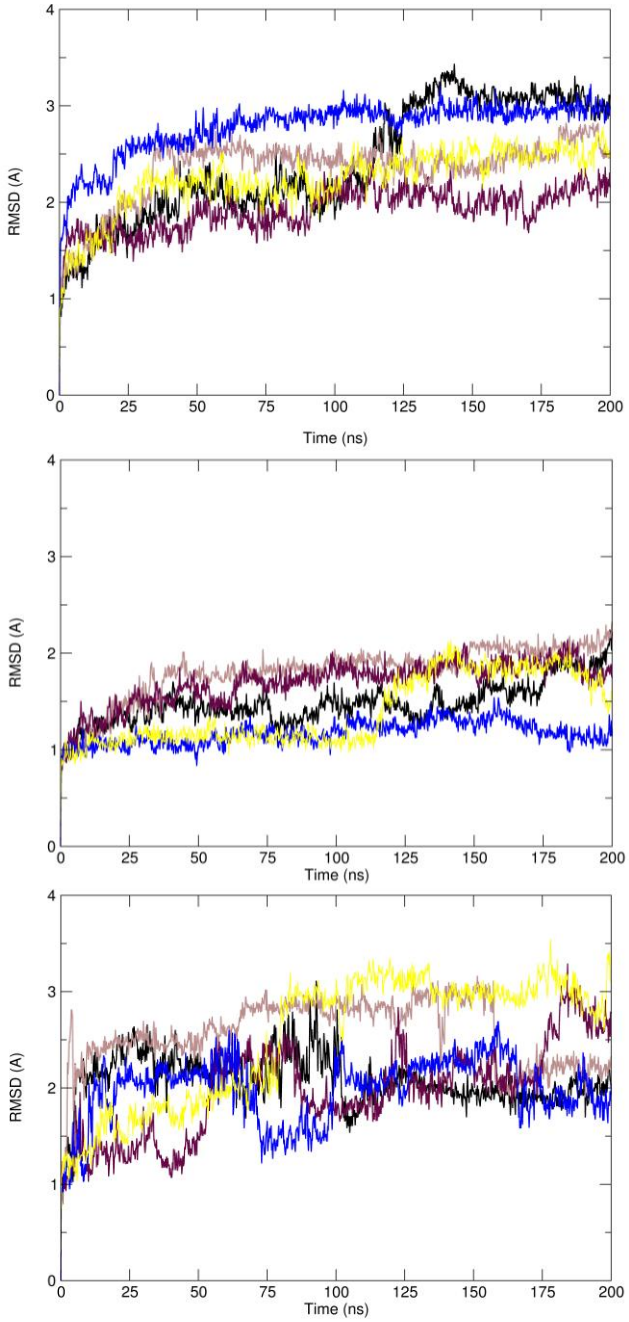

Figure S 2.1: Root mean square deviation (RMSD, 5 x 200 ns overlaid for each system)

Apo enzyme

Ornithine-Arginase complex

ABH-Arginase complex

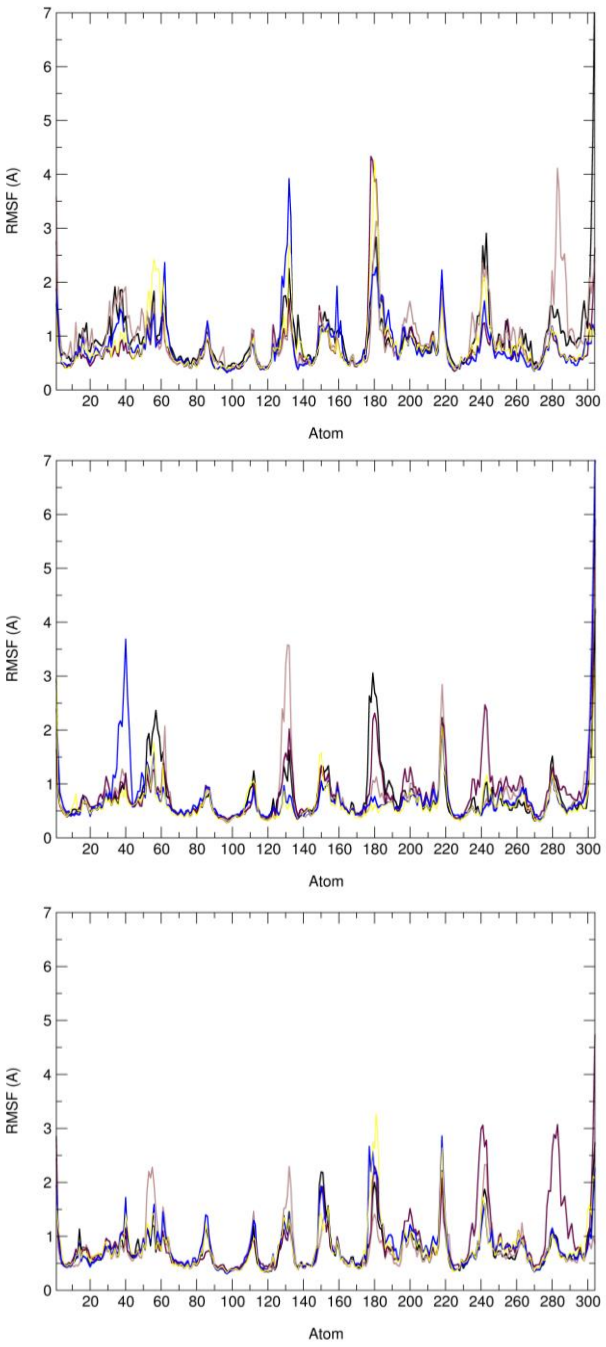

Figure S 2.2: Root mean square fluctuation (RMSF, 5 x 200 ns overlaid for each system)

Analysis of the RMSF can explain how some fluctuations of a particular loop can impact the overall deviations calculated for the complete protein structure. For example, the increase RMSD of the empty enzyme after 100 ns (Figure S 2.1, Apo enzyme, black line) correlates with an unfolding of the C-terminal chain of the protein, as illustrated with the high peak in the RMSF

plot for this same simulation (Figure S 2.2, Apo enzyme, black line). Also, it is interesting to notice that an overall higher deviation from the starting conformation is observed for the ABH-Arginase complex compared to the Ornithine-Arginase complex. Analysis of the fluctuations observed in the regions of the three loops involved in ligand-arginase interactions clearly indicate a similar level of flexibility (RMSF of 3 Å or below for loops around residues 130, 180 and 240). In comparison, these three loops are more dynamic in simulations with the Apo form of Arginase (RMSF reaching 4 Å). This result indicates that the differences in the RMSD between the ABH-Arginase and the Ornithine-Arginase complexes are resulting from movements involving loops at the surface of the protein (such as loops at positions 85, 150 and 280), while both systems show a very similar dynamic of the enzymatic pocket. Analysis of the pocket volume confirms these tendencies simulations with the highest volumes (> 750 Å^3) measured for three out of five 200ns trajectories, while the volume of the same pocket remains below 500 Å^3 with the apo form of arginase (Figure S 2.3).

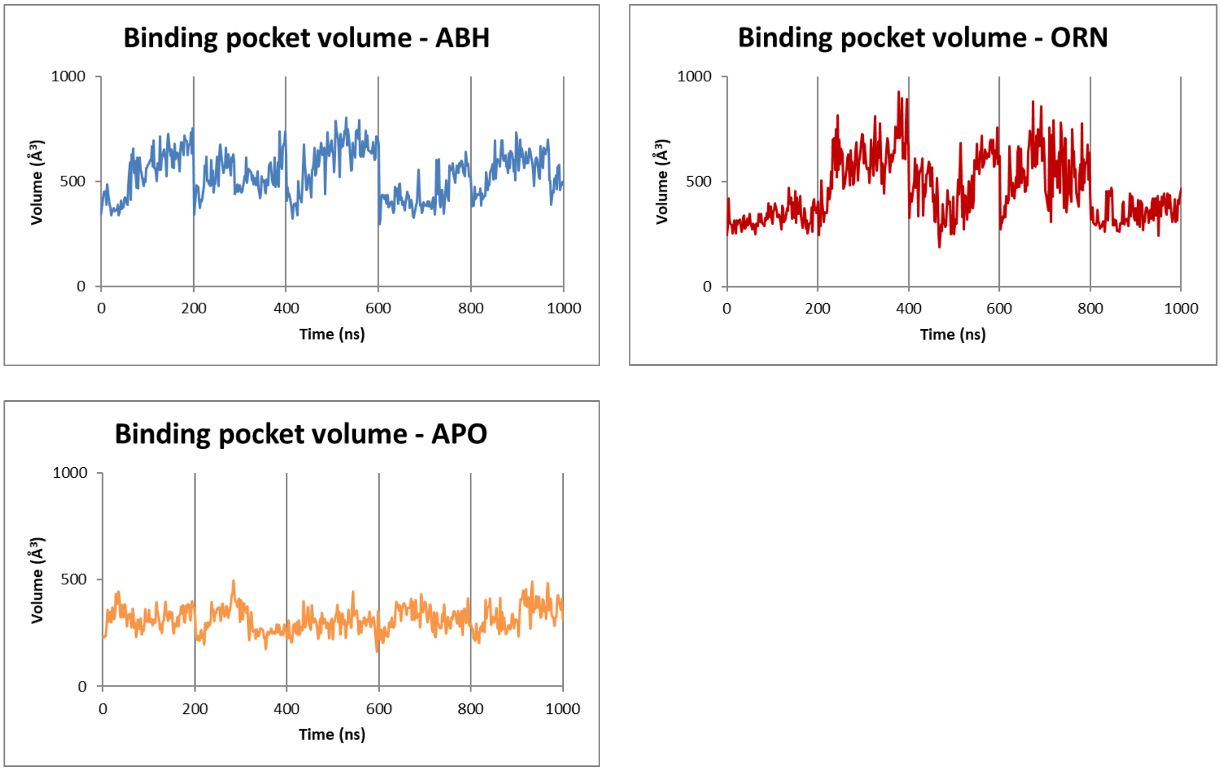

Figure S 2.3: Pocket volume calculation where 200 ns repeats are separated by a vertical line

3. Dynamic pharmacophore

3.1 Arginase-ornithine

The following figures illustrate the analysis of all features represented in the dynophore extracted from 1 µs (5000 frames) of MD simulation trajectory (frames were recorded every 5 ns) with arginase1 and ornithine. Interactions of the same type detected for one single ligand atom were grouped in a so-called superfeature. For each superfeature, all detected interactions were analyzed and reported sequentially (Figure S 3.1.2). Distances between all interaction partners were measured and plotted in a graph with regard to their absolute frequency for each superfeature (Figure S 3.1.3).

Atoms of the ligand involved in counted interactions are referred to by their atom number, as shown in figure S.3.1.1:

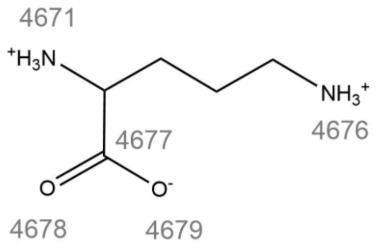

Figure S 3.1.1: 2D-representation of ornithine with numbers for all heavy atoms involved in detected interactions

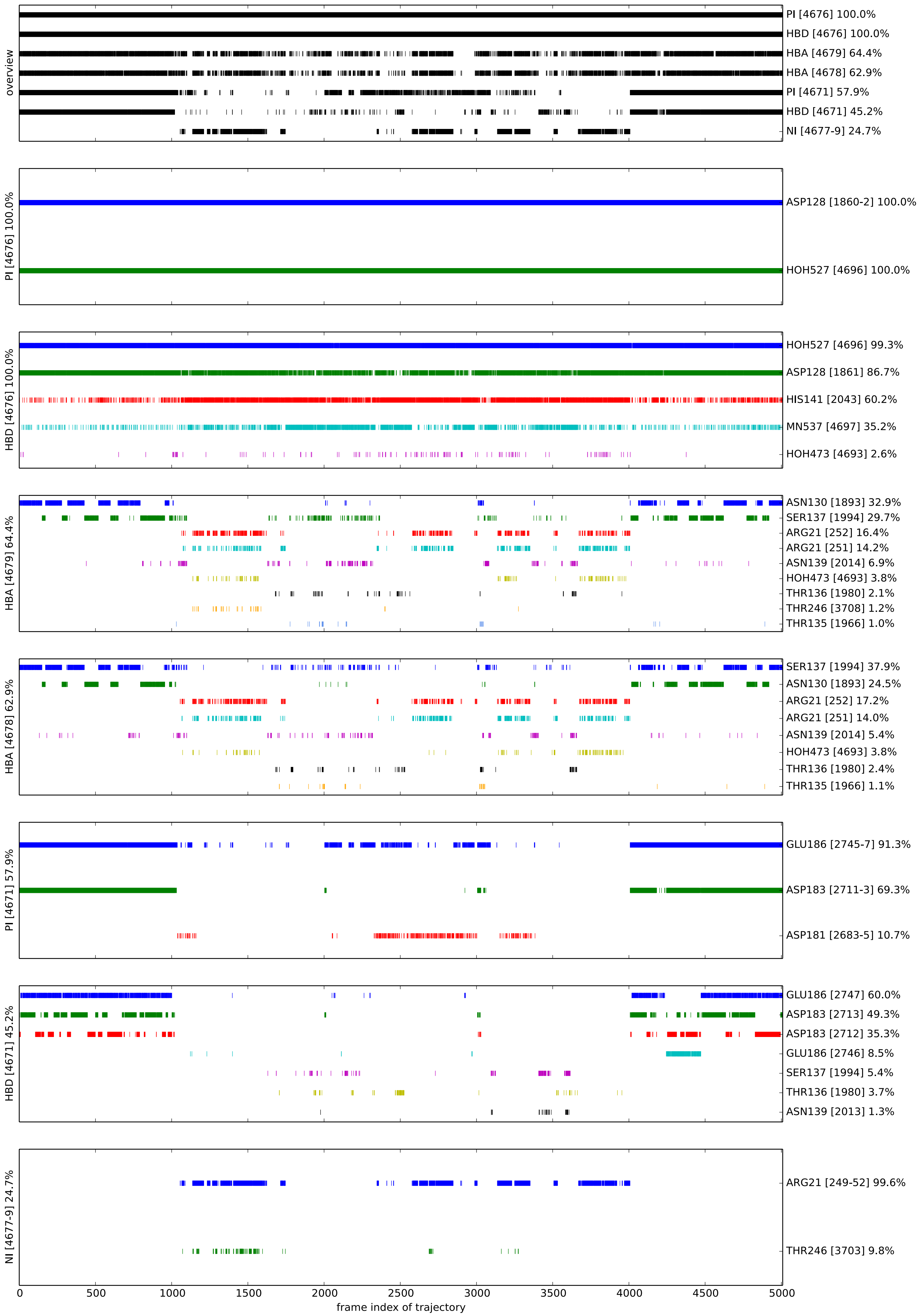

Figure S 3.1.2: Arginase-ornithine - Superfeature occurrence sequences and frequencies (first plot) and interaction occurence sequences and frequencies (plots in color). 5000 Frames represent 1 µs.

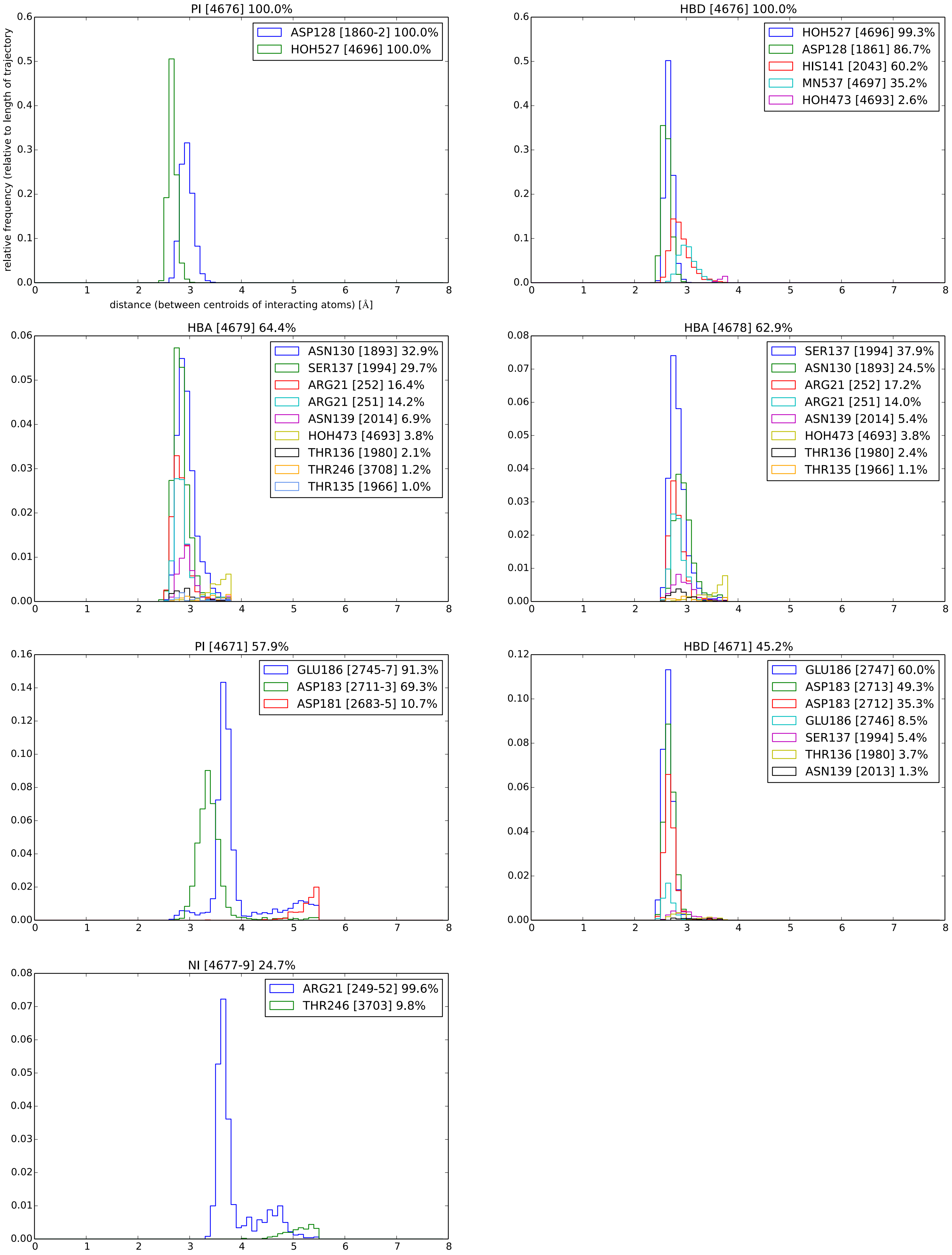

Figure S 3.1.3: Arginase-ornithine - Distance distributions for all interactions detected in each superfeature, where the y-axis represents the relative occurrence frequency (relative to the length of the analyzed trajectory). For clarity in case of rarely detected features, y-axes of the different presented plots are not standardized.

3.2 Arginase-ABH

Using the same method as for the ornithine-arginase complex, the following figures illustrate the analysis of all features detected throughout 1 µs (5000 frames) of MD simulation trajectory (frames were recorded every 5 ns) with arginase1 and inhibitor ABH. All interactions of the same type detected for one single ligand atom are grouped in a *superfeature*. For each superfeature, all interactions are reported sequentially (Figures S 3.2.2-3). Distances between all interaction partners are plotted as described with the ornithine ligand (Figures S 3.2.4-5).

Atoms of the ligand involved in counted interactions are referred to by their atom number, as shown in figure S 3.2.1:

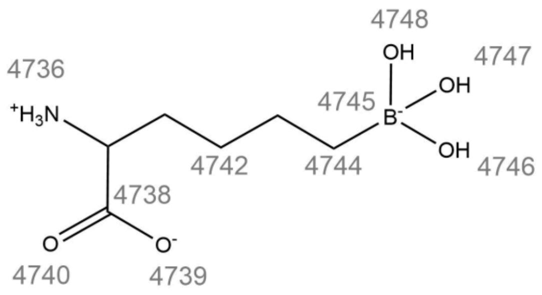

Figure S 3.2.1: 2D-representation of ABH with numbers for all heavy atoms involved in detected interactions.

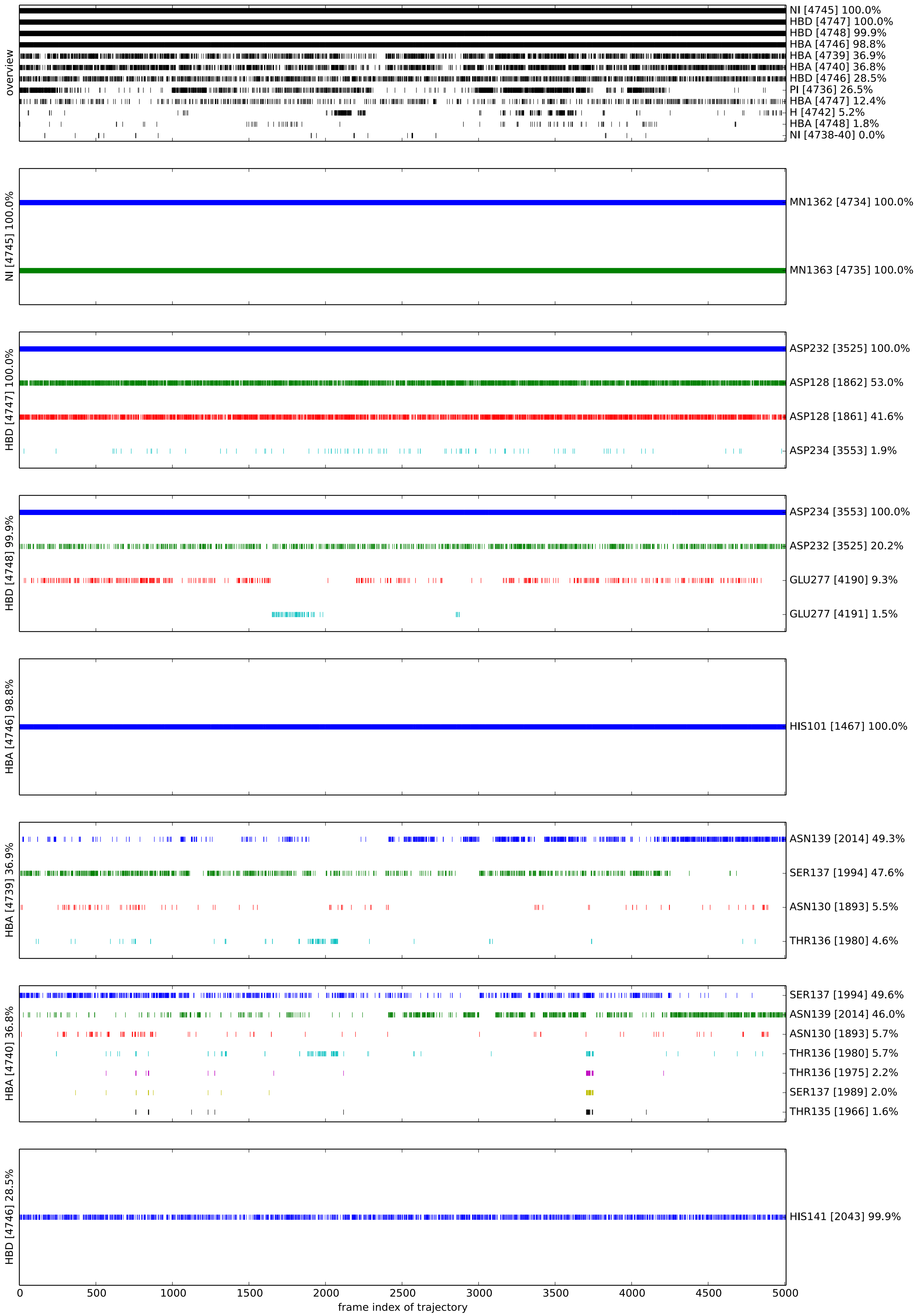

Figure S 3.2.2: Arginase-ABH - Superfeature occurrence sequences and frequencies (first plot) and interaction occurence sequences and frequencies (plots in color) where 5000 frames represent 1 μs (Part 1).

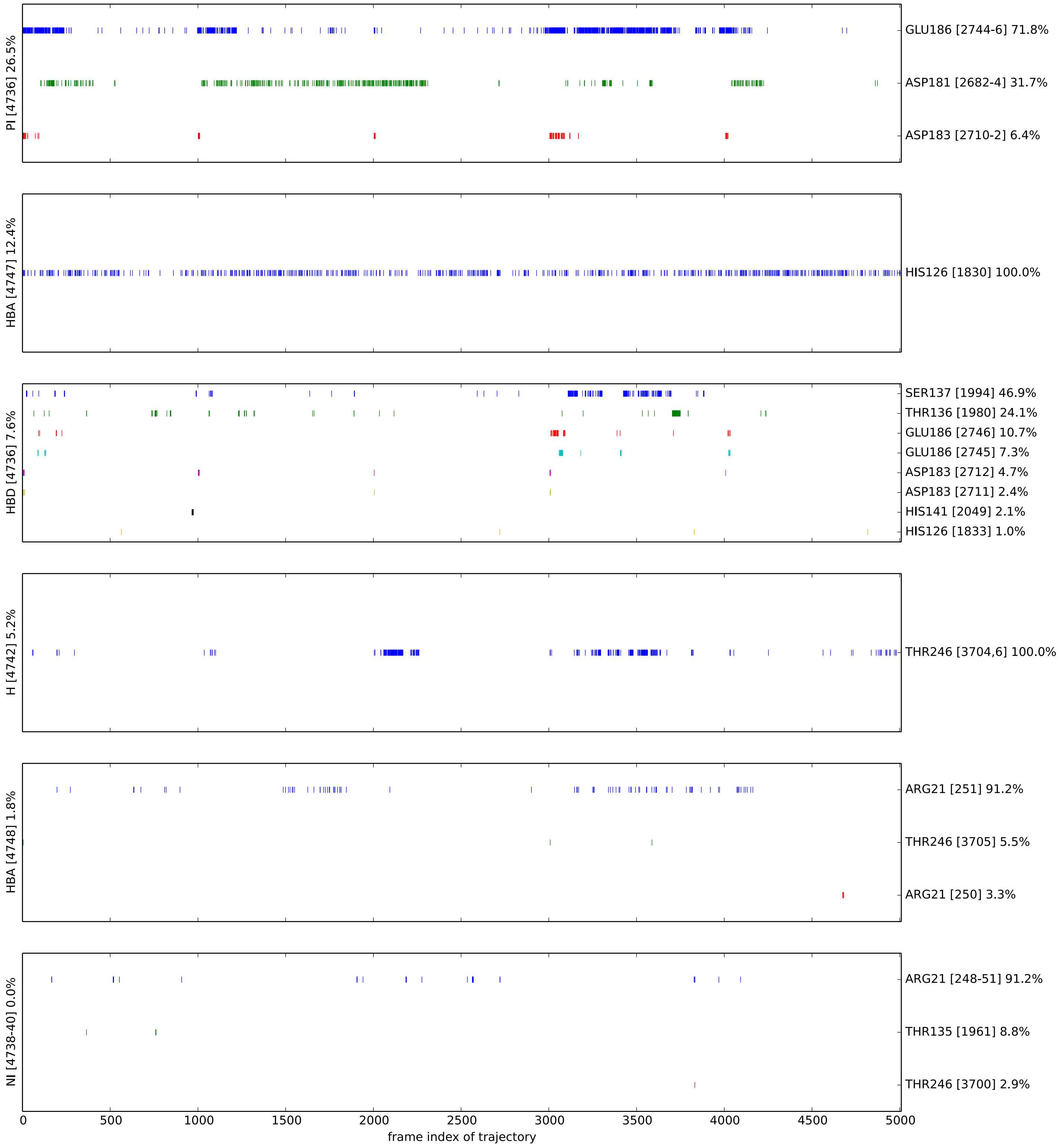

Figure S 3.2.3: Arginase-ABH - Superfeature occurrence sequences and frequencies (first plot) and interaction occurence sequences and frequencies (plots in color) where 5000 frames represent 1 μs (Part 2).

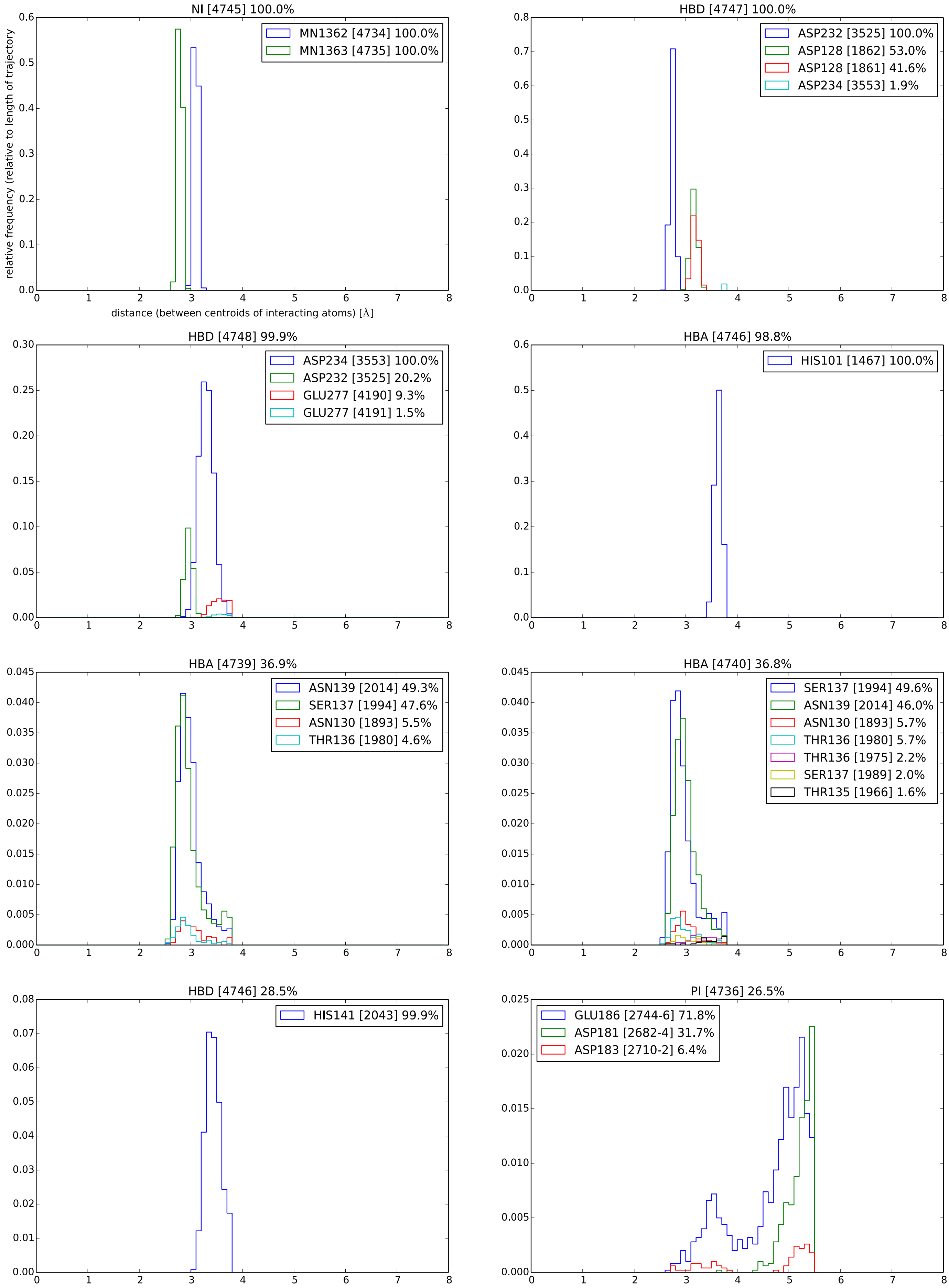

Figure S 3.2.4: Arginase-ABH - Distance distributions for all interactions detected in each superfeature, where the y-axis represents the relative occurrence frequency (relative to the length of the analyzed trajectory). For clarity in case of rarely detected features, y-axes of the different presented plots are not standardized (Part 1).

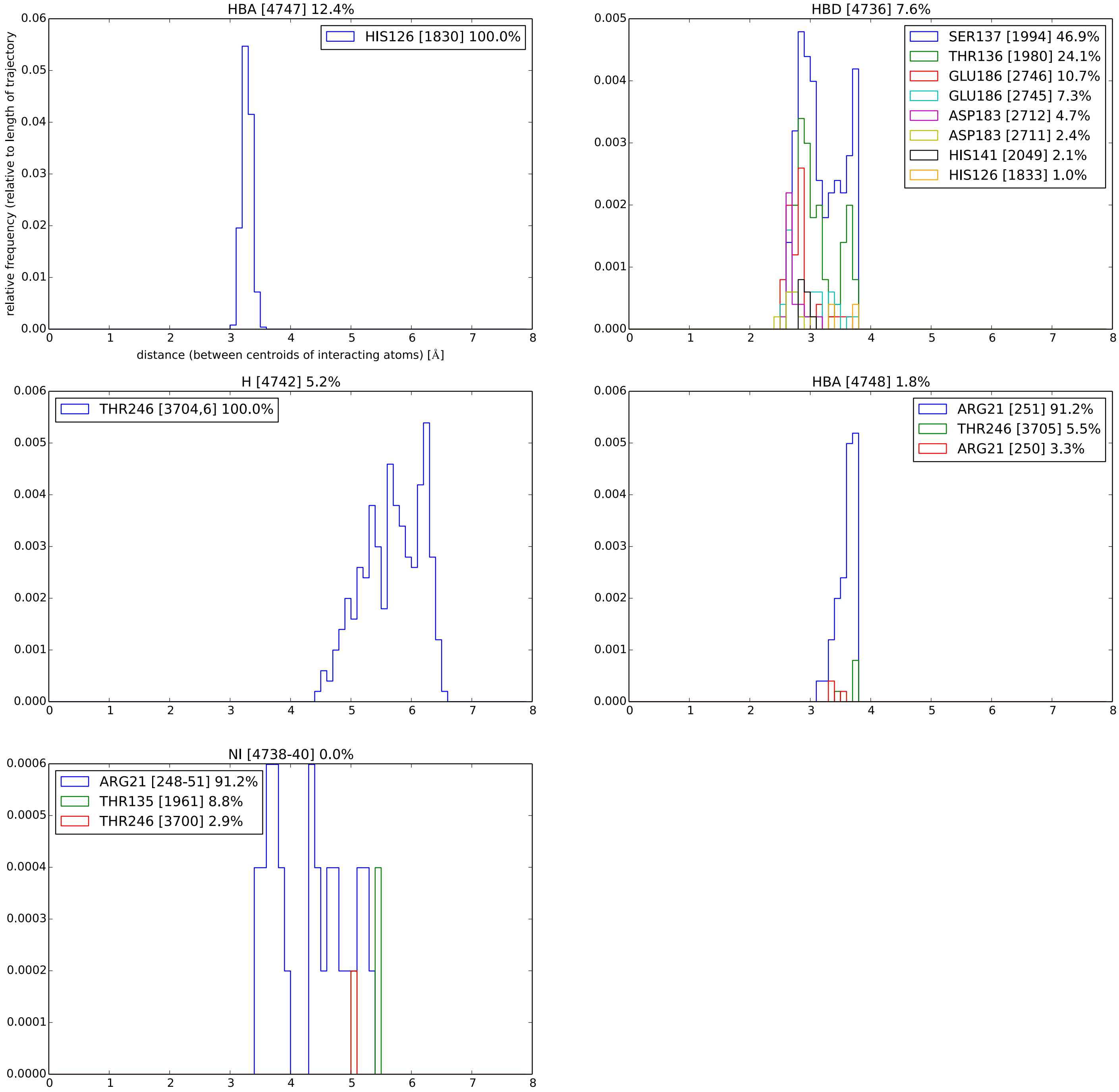

Figure S 3.2.5: Arginase-ABH - Distance distributions for all interactions detected in each superfeature, where the y-axis represents the relative occurrence frequency (relative to the length of the analyzed trajectory). For clarity in case of rarely detected features, y-axes of the different presented plots are not standardized (Part 2).

4. Fragments testing

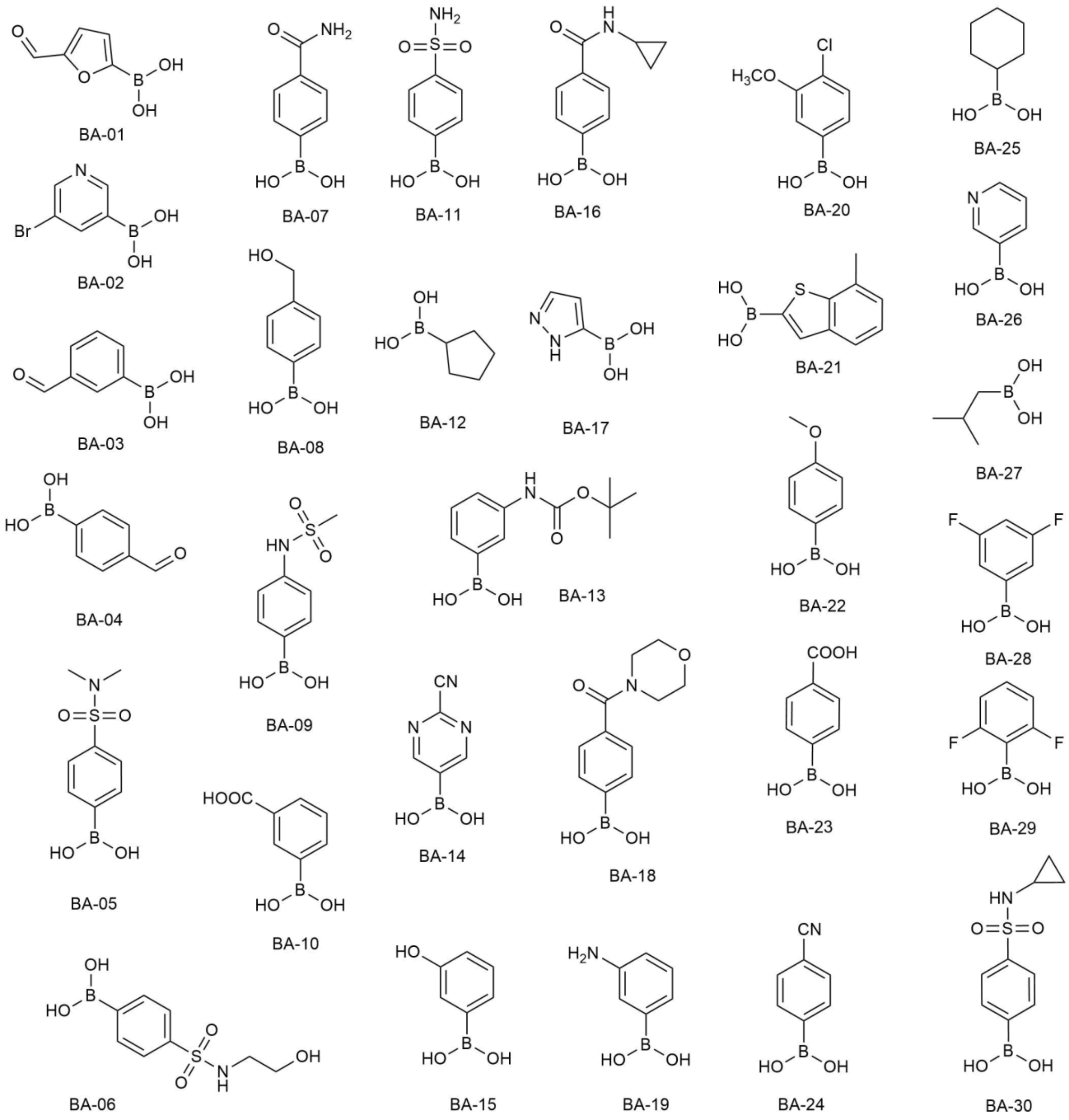

Figure S 4.1: Chemical structure of all tested boronic acid fragments

An inhibitory curve was also measured for the most potent compounds, sulfamoylbenzene-boronic acid (BA-11) and cyclohexylboronic acid (BA-25), showing enzymatic activity reduced by half at an inhibitor concentration of 0.2 mM for BA-25 (IC_{50} calculated from an extrapolated curve and therefore approximate, as no inhibitory potency could be measured at higher concentrations due to solubility issues).

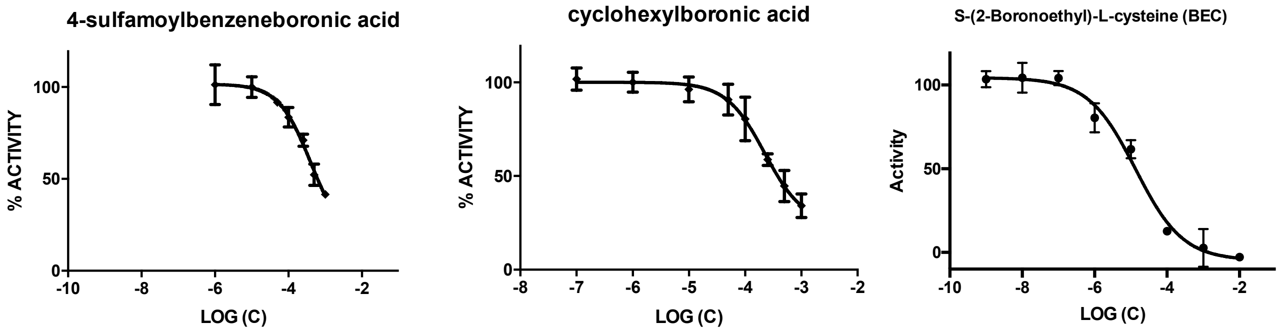

Figure S 4.2: IC_{50} measurements for boronic acid fragments BA-11 (left) and BA-25 (centre) as well as the reference inhibitor BEC (right)

5. Computational study of the BA-25 binding mode

A dynophore was created from MD simulations of arginase in complex with BA-25 (Figure S 5.1). Following the same method as introduced previously, figures S5.3-5 illustrate the analysis of all features represented in the dynophore generated from 1 µs (5000 frames) of MD simulation trajectory (frames were recorded every 5 ns). All interactions of the same type detected for one single ligand atom are grouped in a *superfeature* (Figure S 5.3). For each superfeature, all interactions are reported sequentially (Figure S 5.4). Distance distributions are plotted for all interactions relatively to their absolute frequency (Figure S 5.5).

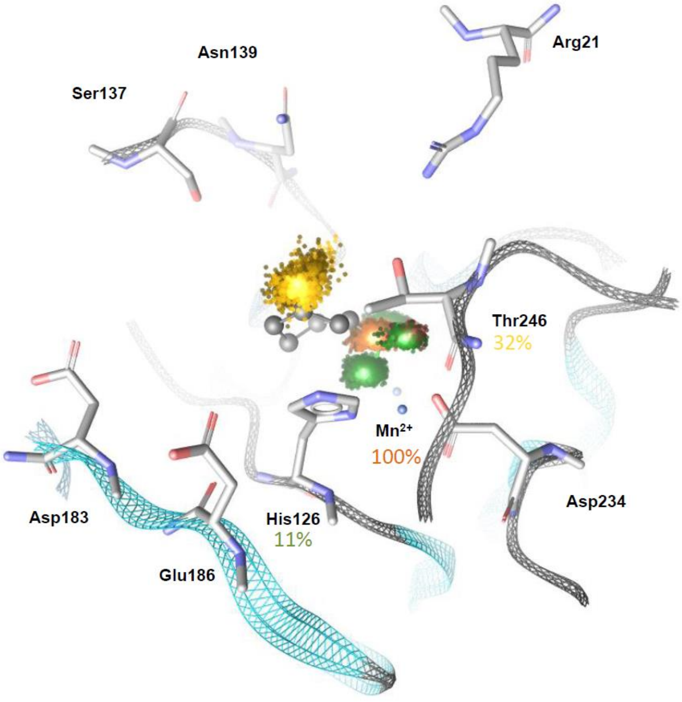

Figure S 5.1: Dynophore for the arginase-BA-25 complex (orange for negative ionizable features, green for H-bond donors, blue for positive ionizable moieties and yellow for hydrophobic contacts).

Atoms of the ligand involved in counted interactions are referred to by their atom number, as shown in figure S 5.2:

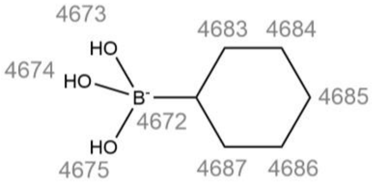

Figure S 5.2: Compound BA-25 with numbers for all heavy atoms

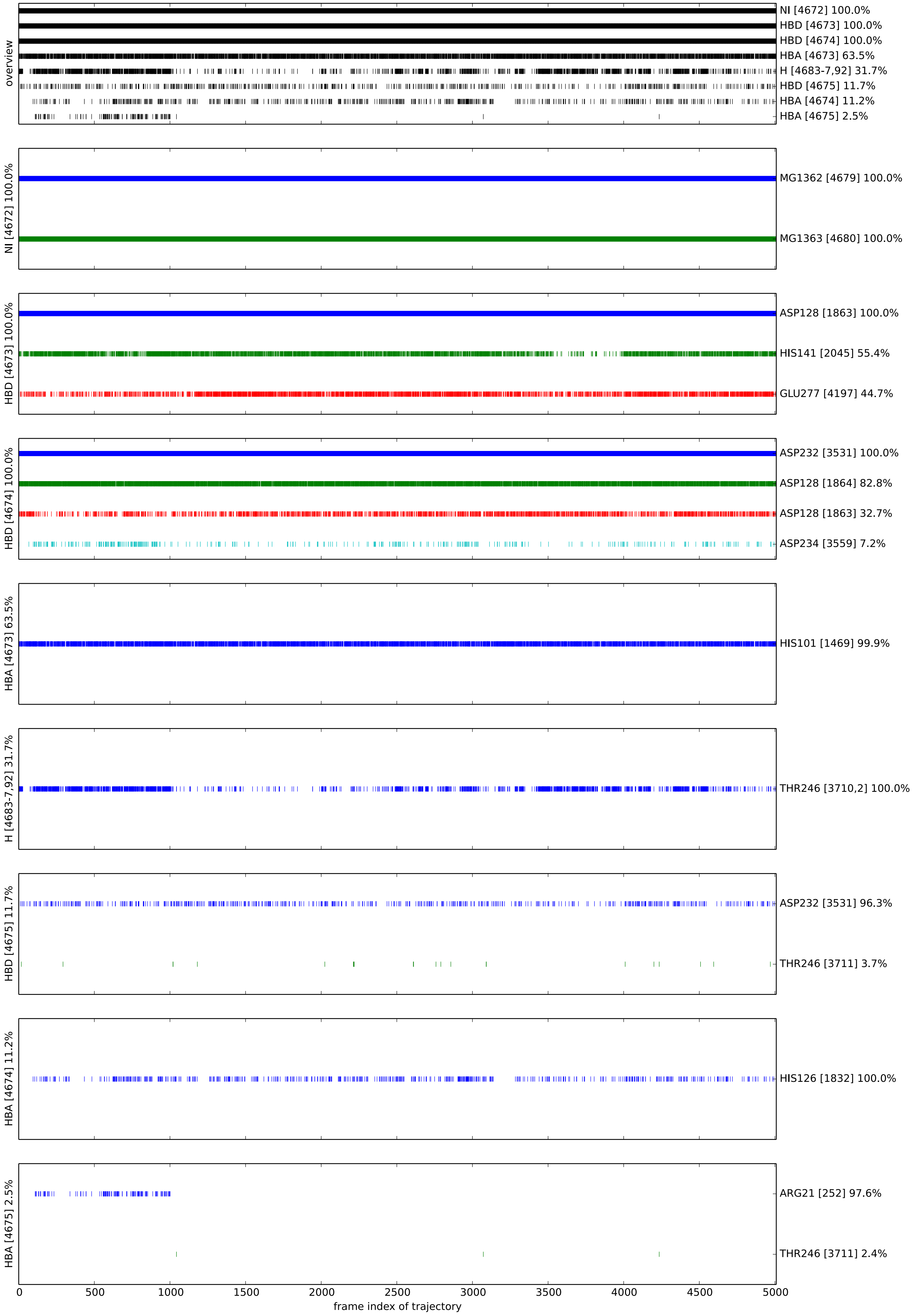

Figure S 5.3: Arginase-BA-25 - Superfeature occurrence sequences and frequencies (first plot) and interaction occurence sequences and frequencies (plots in color). 5000 Frames represent 1 μs.

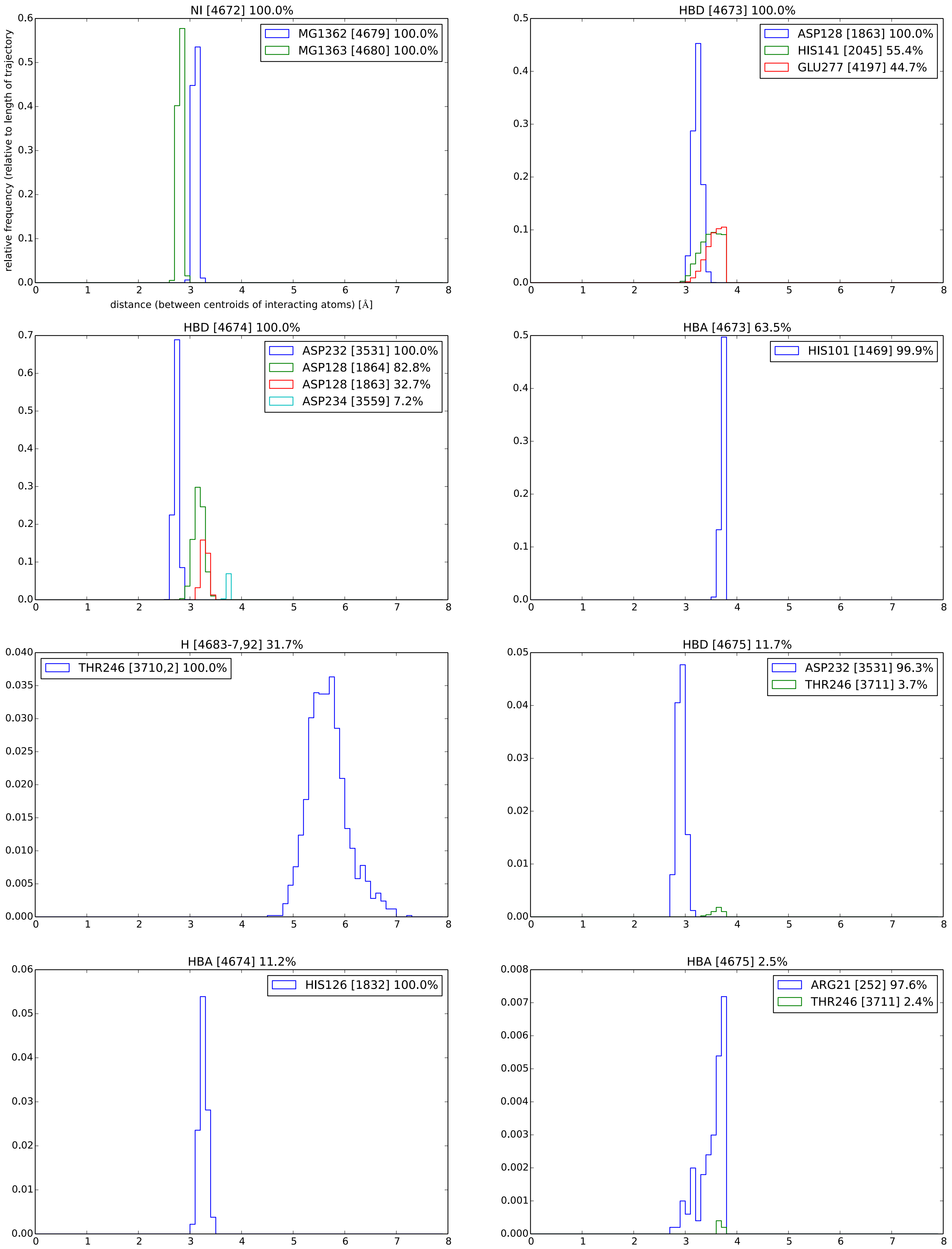

Figure S 5.4: Arginase-BA-25 - Distance distributions for all interactions detected in each superfeature, where the y-axis represents the relative occurrence frequency (relative to the length of the analyzed trajectory). For clarity in case of rarely detected features, y-axes of the different presented plots are not standardized.

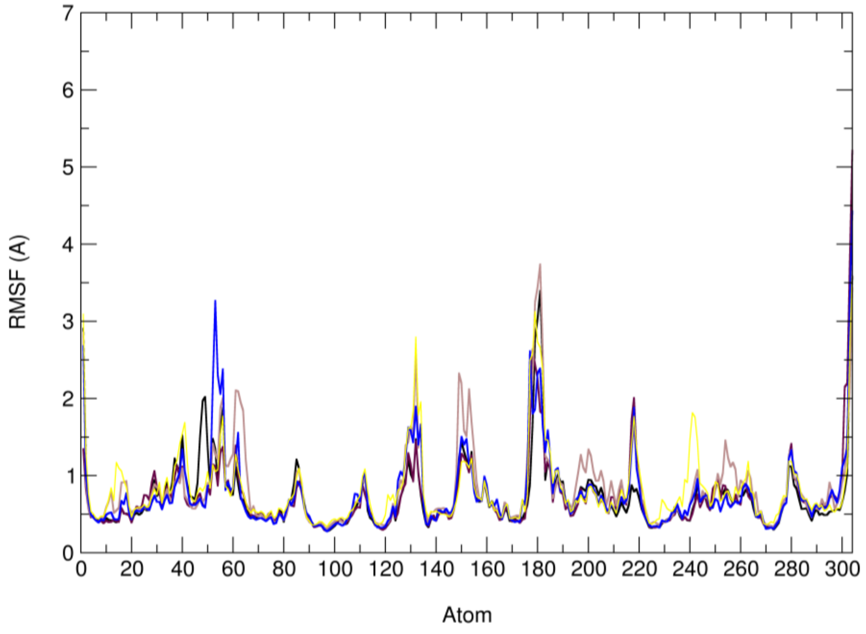

Figure S 5.5: Root mean square fluctuations (RMSF) overlay from five 200ns MD simulations

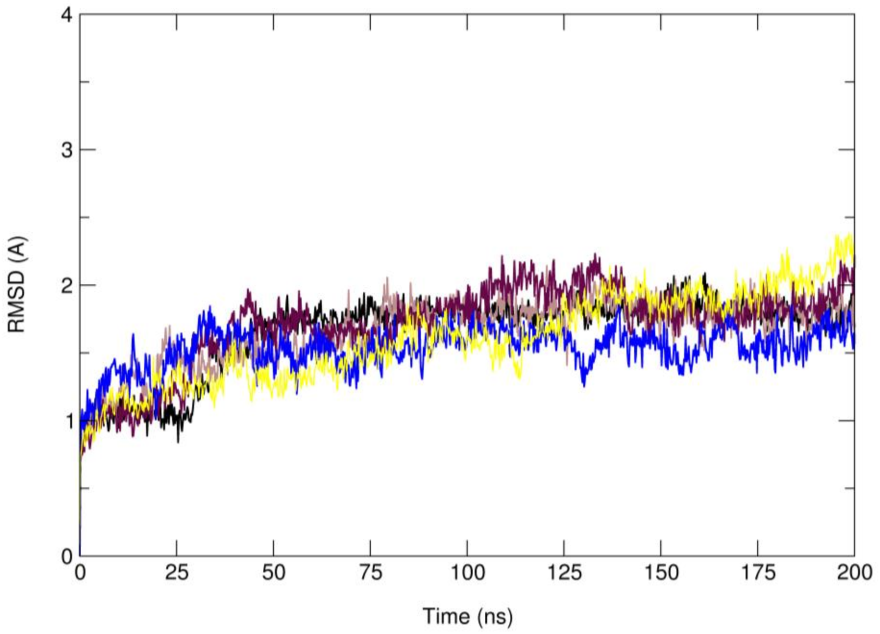

Figure S 5.6: Root mean square deviations (RMSD) overlay from five 200ns MD simulations

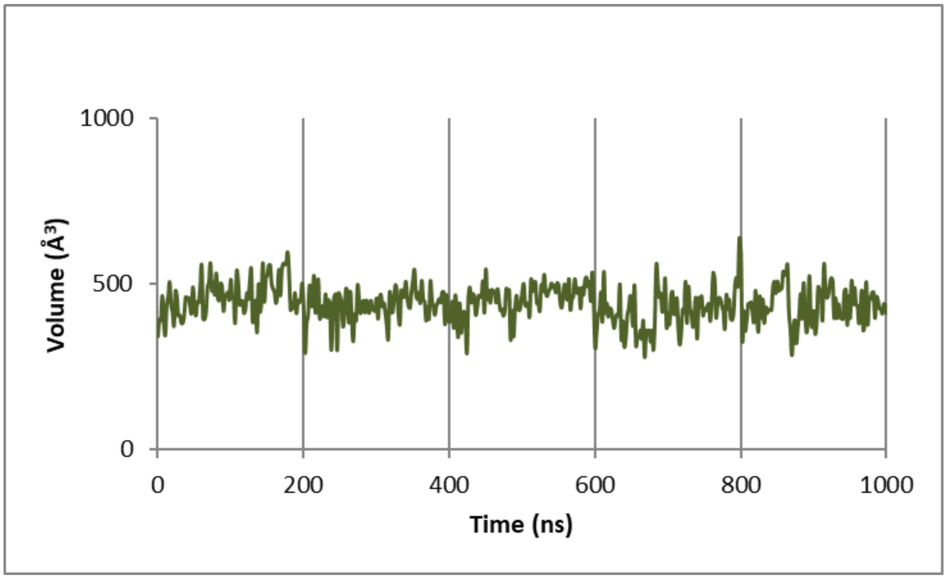

Figure S 5.7: Pocket volume calculation where 200 ns repeats are separated by vertical lines
